# Climate-denying rumor propagation in a coupled socio-climate model: Impact on average global temperature

**DOI:** 10.1371/journal.pone.0317338

**Published:** 2025-01-16

**Authors:** Athira Satheesh Kumar, Chris T. Bauch, Madhur Anand

**Affiliations:** 1 Department of Applied Mathematics, University of Waterloo, Waterloo, Ontario, Canada; 2 School of Environmental Science, University of Guelph, Guelph, Ontario, Canada; Roma Tre University: Universita degli Studi Roma Tre, ITALY

## Abstract

Individual attitudes vastly affect the transformations we are experiencing and are vital in mitigating or intensifying climate change. A socio-climate model by coupling a model of rumor dynamics in heterogeneous networks to a simple Earth System model is developed, in order to analyze how rumors about climate change impact individuals’ opinions when they may choose to either believe or reject the rumors they come across over time. Our model assumes that when individuals experience an increase in the global temperature, they tend to not believe the rumors they come across. The rumor rejectors limit their CO_2_ emissions to reduce global temperature. Our numerical analysis indicates that, over time, the temperature anomaly becomes less affected by the variations in rumor propagation parameters, and having larger groups (having more members) is more efficient in reducing temperature (by efficiently propagating rumors) than having numerous small groups. It is observed that decreasing the number of individual connections does not reduce the size of the rejector population when there are large numbers of messages sent through groups. Mitigation strategies considered by the rejectors are highly influential. The absence of mitigative behavior in rejectors can cause an increase in the global average temperature by 0.5°C. Our model indicates that rumor propagation in groups has the upper hand in controlling temperature change, compared to individual climate-denying propagation.

## 1 Introduction

Sustainability challenges have spanned multiple systems such as forest degradation [1–3], air pollution [5–7] and water systems [8–10], and accelerated anthropogenic global warming. Since the Industrial Revolution, humans have been emitting increasing amounts of greenhouse gases (GHG) into the Earth’s atmosphere, leading to anthropogenic global warming. The Intergovernmental Panel on Climate Change (IPCC) reports [4, 11] outline the social transitions required to limit the increase in the average global temperature to between 1.5°C and 2°C. Lifestyle and behavioral changes like reduction in household and industrial energy consumption, switching to renewable energy, and changes in diet can assist in achieving this goal [12–14]. Climate models couple Earth system components like ocean and terrestrial systems with atmospheric dynamics, in order to project future temperature, precipitation, and other variables [15–20]. These model dynamics respond to atmospheric GHG concentration, the roots of which are human activities like fossil fuel usage and deforestation. These models typically assume a fixed trajectory for future GHG emissions as a model input, based on assumptions about socio-economic development. However, GHG emissions are not fixed but rather they increasingly respond to climate change, as a result of the response from human populations and institutions [21–23].

Human perceptions of climate change, variation in individual behavior driving climate change, and human responses and adaptation strategies are recognized as the three key aspects of human behavioral changes in response to climate change [23]. Human beings recognize and adapt to climate change based on personal experience [24, 25], for instance, individuals living in places with increased temperatures are more likely to perceive climate change than others [22]. This observation suggests that human behavior and climate change are connected into a single coupled human-environment system [26, 27]. Changes in individual behaviors could also contribute to climate change mitigation strategies [27, 28]. Behavioral change alone can lead to significant reduction in emissions [29]. With monetary incentives and feedback, around 0.35 GTCO_2_ per year reduction in emission levels can be observed from the residential sector alone [30, 31]. A study regarding potential behavioral changes on climate change in the European Union further establishes the importance of including human behavior: changes to dietary habits, mobility, and household can reduce the emission levels by 6% to 16% leading to a decrease of 13.5% to 30% reduction mitigation costs [32]. These findings underscore the importance of population behavioral transitions in climate change mitigation, which leads to substantial reduction in emissions.

Coupled socio-climate models have been gaining importance due to the growing recognition of the role of social processes. Such models have demonstrated the enormous influence of social norms and social learning on temperature projections [27, 33]. These models act as tools to understand the feedback loops between climate and human systems. Several behavioral theories could be potentially used in the coupled model [34–36]. Models involving frameworks for representing processing, spreading, and behavioral responses considering the heterogeneous nature of society have also been developed recently. Qualitative insights on socio-climate models portray the importance of social learning, and the associated cost for mitigation [27]. By combining an Earth System Model with a social dynamics model, Bury et al. [27] observed that increasing social learning and reducing mitigation costs is the most efficient way to reduce global temperature anomalies. These models also help to analyze how different groups in a heterogeneous population respond and evolve [37].

Several models have coupled climate dynamics and economic processes. For instance, integrated assessment models (IAMs) couple climate dynamics and economic processes by incorporating economic feedback in agriculture and energy expenditures [38, 39]. Dynamic Integrated Climate Economy (DICE) models couple climate change and economies with a focus on mitigation costs [40, 41]. However, human attitudes towards climate change could be influenced by the social, religious, or political views in the population [42, 43]. Therefore, models that also include other relevant human processes–like social dynamics–are required for a more comprehensive picture. Machine learning models are also gaining popularity in studying climate systems to predict temperature and precipitation [44].

Various rumor propagation models have been developed and analyzed in different domains. Rumor propagation models inspired by compartmental models are not new [45–47]. Due to the similarities between disease spread and rumor propagation in a population, rumor propagation models are sometimes constructed using mathematical epidemiology [48]. They also provide deeper insights on various factors that helps in understanding how to mitigate the effects of rumors. But most of these models focus on individual rumor dynamics. There are very few models that address propagation of rumors in groups [49]. Individual rumor propagation occurs when an individual spreads rumors by sharing them with other individuals whereas, in group propagation, an individual propagates the rumor to an entire group of individuals, such as via social media. Various aspects of group and individual rumor propagation have the power to affect individuals’ attitudes toward climate change. Most of the population has only a superficial knowledge of climate change [50]. Perhaps as a result, rumors about climate change–especially climate misinformation–are common and there are empirical studies and statistical models that deal with myths and fake news regarding climate change [51, 52].

Several coupled models deal with climate change and various aspects of human behavior, integrating features such as imitation dynamics and social norms [27, 37]. However, previous coupled social-climate models have not included rumor propagation, despite the fact that rumours which deny climate change are a major reason for individuals and groups not acknowledging the urgency of the climate emergency, and thus not taking effective mitigation strategies [53, 54]. Here, a coupled socio-climate model is developed where rumor propagation spreads through a mathematical approximation of a social network. Our objective is to demonstrate a simple yet novel way of modeling interactions between rumor spread as an contagious processes and long-term climate dynamics to gain insights into potential social-climate dynamics. A simple Earth system model is used to capture temporal dynamics of the global temperature anomaly, and our rumor propagation model is correspondingly simple. Although social-climate systems are complex, starting with a simple model can be useful to illustrate concepts, discover new dynamical regimes, and suggest a framework for future, more sophisticated social-climate models.

## 2 Methods

A mathematical model linking climate-denying rumor propagation (including group dynamics) in heterogeneous networks with climate dynamics is proposed. The coupled rumor-climate change model is proposed by adding the climate dynamics into an existing rumor propagation model [49] in social networks by relating the human responses to rumors based on the range of climate change they experience. In this extension, the rumors are assumed to be false information about climate change. The model is developed by coupling the rumor-spreading model with an Earth System Model (ESM) to analyze the dynamics of the coupled system, in which the rumor propagation about climate change begins in 2021. The model considers a heterogeneous rumor model since real-life networks, including social networking sites, are heterogeneous in nature. This is done by incorporating emissions (mainly CO_2_, an important greenhouse gas) and temperature into the model. The rumor model has two components: individual propagation and group propagation. The model helps us interpret how the parameters involved in rumor propagation influence emission levels and temperature change and how changes in temperature levels influence human attitude towards the rumors about climate change. The rumor-climate change dynamics are formulated such that when individuals understand that the rumors are false, they try to mitigate climate change (S1 Fig). Similarly, individuals who experience climate change, through changes in temperature, believe that the rumors are not true.

### 2.1 Rumor propagation model

An existing rumor propagation model involving group propagation in heterogeneous networks is used [49]. Each node represents either an individual or a group in the social network, and the edges are connections between the individuals or between individuals and the groups they belong to. Each individual is assumed to be in one of the following states: *s*_*k*_ denotes the proportion of susceptible individuals who are susceptible to the rumor about climate change but are not exposed to the rumor, *h*_*k*_, hesitator is the individual who is exposed to the rumor but hesitates to believe or reject the rumor. If they accept it, they propagate the rumor. Otherwise, they reject it. *i*_*B*_ is the proportion of believers who believe climate change is not real and share the rumor individually and in groups, and *i*_*R*_ is the proportion of rejectors who reject the rumor, believing that climate change is real, and do not forward the rumor.

The equations for the rumor propagation involve ordinary differential equations and are as follows [49]:

$$\begin{array}{c} \begin{array}{cc} s_{k}^{\prime }\left(t\right) & =B-\beta \bar{c}ks_{k}\left(t\right)\frac{\sum_{j}\left(p\left(j|k\right)\right)}{p_{j}}i_{B_{j}}\left(t\right)+\alpha \bar{c}k\frac{\sum_{j}\left(p\left(j|k\right)\right)}{p_{j}}i_{B_{j}}\left(t\right)+\gamma \bar{c}ki_{R_{k}}\left(t\right)-\bar{G}\bar{m}\bar{d}\varsigma s_{k}\left(t\right)\\ & i_{B_{k}}\left(t\right)+\bar{G}\bar{m}\bar{d}\sigma i_{R_{k}}\left(t\right)+\bar{G}\bar{m}\bar{d}\rho i_{B_{k}}\left(t\right)-\mu s_{k}\left(t\right) \end{array} \end{array}$$

$$\begin{array}{c} \begin{array}{cc} h_{k}^{\prime }\left(t\right) & =\beta \bar{c}s_{k}\left(t\right)k\frac{\sum_{j}\left(p\left(j|k\right)\right)}{p_{j}}i_{B_{j}}\left(t\right)-\left(\eta \left(T\right)+\varepsilon \right)\bar{c}k\frac{\sum_{j}\left(p\left(j|k\right)\right)}{p_{j}}h_{j}\left(t\right)+\bar{G}\bar{m}\bar{d}\varsigma s_{k}\left(t\right)i_{B_{k}}\left(t\right)-\\ & \bar{G}\bar{m}\bar{d}\omega \left(T\right)h_{k}\left(t\right)-\bar{G}\bar{m}\bar{d}\zeta h_{k}\left(t\right)-\mu h_{k}\left(t\right) \end{array} \end{array}$$

$$\begin{array}{c} \begin{array}{cc} i_{B_{k}}^{\prime }\left(t\right) & =\eta \left(T\right)\bar{c}k\frac{\sum_{j}\left(p\left(j|k\right)\right)}{p_{j}}h_{j}\left(t\right)-\alpha \bar{c}k\frac{\sum_{j}\left(p\left(j|k\right)\right)}{p_{j}}i_{B_{j}}\left(t\right)+\bar{G}\bar{m}\bar{d}\omega \left(T\right)h_{k}\left(t\right)-\bar{G}\bar{m}\bar{d}\rho \\ & i_{B_{k}}\left(t\right)-\mu i_{B_{k}}\left(t\right) \end{array} \end{array}$$

$$\begin{array}{c} i_{R_{k}}^{\prime }\left(t\right)=\varepsilon \bar{c}k\frac{\sum_{j}\left(p\left(j|k\right)\right)}{p_{j}}h_{j}\left(t\right)-\gamma \bar{c}ki_{R_{k}}\left(t\right)+\bar{G}\bar{m}\bar{d}\zeta h_{k}\left(t\right)-\bar{G}\bar{m}\bar{d}\sigma i_{R_{k}}\left(t\right)-\mu i_{R_{k}}\left(t\right) \end{array}$$ (1.1)

Social media features, like entering and leaving the network at any stage, are considered in this model. Individuals joining the network always fall into the susceptible category, and the number of individuals joining the network per unit of time is *B*. An individual can exit the network at any time, and hence the exiting rate at which an individual exits the network *μ* is included in each compartment. Since both individual and group propagation are considered, both parameters are considered separately. *β* is the hesitating probability with which a susceptible individual becomes a hesitator individual, whereas the hesitating probability in groups is *ς*. The probability that a hesitator individual believes the rumor is *η*, and in groups, this probability is *ω*. Similarly, a hesitator individual rejects the rumor with probability *ϵ*; in groups, this probability is *ζ*. A forgetting mechanism is also included in this model, along with believing and rejecting mechanisms. Individuals will forget the rumor and their opinion about it, as time passes and revert to the initial susceptible state. The probability with which the believer and rejector individuals forget the rumor is *α* and *γ*, respectively. The term *p*(*j*|*k*) denotes the probability with which an edge with degree *k* connects to a node with degree *j*. The respective rates in groups are considered as *ρ* and *σ*. Apart from the probabilities, the average rate of individual contact is $\bar{c}$, and the average rate at which messages are transmitted per group is $\bar{d}$. The average network degree is *k* with *X* groups having *m* members on average, with the average degree for the group for an individual being $\bar{G}$.

### 2.2 Earth system model

A relation between emissions and temperature is required to understand the increase in temperature due to emissions. Earth system models (ESMs) describe climate-relevant Earth processes and how they influence climate dynamics. A simple existing Earth system model is used for this purpose [55]. An austere climate–carbon-cycle model for understanding the coupled climate-carbon–cycle system defines the global mean surface temperature anomaly (controlled by the upper ocean) as follows:

$$\begin{array}{c} \frac{dT}{dt}=\frac{qF-T}{d} \end{array}$$ (1.2)

$$\begin{array}{c} F=\frac{F_{2x}}{ln(2)}ln\left(\frac{C_{atm}}{C_{0}}\right) \end{array}$$ (1.3)

where $F=\frac{F_{2x}}{ln(2)}ln\left(\frac{C_{atm}}{C_{0}}\right)$ is the CO2 radiative forcing and C_0_ is the pre-industrial atmospheric CO_2_ level and *C*_*atm*_ is the atmospheric CO_2_ level at the time point [55]. *F*_2x_ is the forcing due to CO_2_ doubling, *q* is the thermal adjustment of the upper ocean, and *d* is the thermal equilibrium for the upper ocean. The global mean surface temperature anomaly helps in understanding the change in temperature that has occurred over the years and projecting future changes concerning the emission levels (S1 Table). This ESM has been used to calculate the temperature anomaly under different emissions scenarios [56].

### 2.3 Coupled rumor—Climate change model

The rumor propagation model in section 1.1 is modified by incorporating climate dynamics. The coupling is accomplished by making the individual believing probability *η* and group believing probability *ω* a function of the change in temperature, *T*, defined as:

$$\begin{array}{c} \eta \left(T\right)=\eta_{0}e^{-vT} \end{array}$$ (1.4)

$$\begin{array}{c} \omega \left(T\right)=\omega_{0}e^{-vT} \end{array}$$ (1.5)

The parameters in the system Eq 1.1 are described in S1 Table. According to Eqs 1.4 and 1.5, the believing probability increases or decreases in response to the change in the temperature (*T*). As *T* increases, *η* and *ω* decrease: fewer individuals are convinced by the rumors since they experience the temperature change. This will decrease the believing probability and increase the rejecting probability.

The deviation of the global atmospheric CO_2_ levels from the pre-industrial levels, *C*, is defined as:

$$\begin{array}{c} \frac{dC}{dt}=\{\begin{array}{cc} E(t) & t\leq 2021\\ E_{2021}*exp\left(-\Psi \sum_{k}i_{R_{k}}\right)-\delta C & t>2021 \end{array} \end{array}$$ (1.6)

where *E*(*t*) is the anthropogenic emission data for each year until 2021, Ψ is a constant value at which the rejectors limit their emissions, which is any mitigation strategies considered by the rejector to reduce their emissions, like switching to renewable energy like solar energy for power consumption, using electric or hybrid vehicles, insulating walls during construction, etc. *E*_2021_ is the baseline carbon dioxide emissions level in the absence of rejectors (amount in 2021). According to Eq 1.6, CO_2_ emissions fall when the rejector population increases exponentially at a constant value Ψ. Apart from this, emission gets naturally dissipated due to various natural processes (sinks like ocean, land, and plants), and this natural dissipation rate is *δ*.

Usually, the time scale of rumor propagation and climate change is not comparable. Rumor propagation might last for weeks or months, whereas climate change takes place over decades. To overcome this issue, the long-term susceptibility and transmission of individuals is modeled to a collection of multiple related rumors. This means that an individual who is a believer (rejector), believes (rejects) various rumors that come up over time, and not just a single rumor.

### 2.4 Analysis

Time series, univariate, and bivariate (parameter plane) sensitivity analysis is used to understand the model’s behavior dynamics and parameter sensitivity. The baseline parameter values for the model were generated using Approximate Bayesian Computation (ABC) [57, 58], and the baseline climate parameters are obtained from the ESM [55], and the Global Carbon Budget [56]. The parameter range considered for sensitivity analysis depends on the upper and lower bound of the model parameters. These bounds are chosen in accordance with the nature of the parameter. For example, the parameters representing the probability values are considered between 0 and 1, and hypothetical model parameters are given broad upper and lower bounds, specified in S1 Table. The analysis has been carried out over 180 years which began when the rumor propagation emerged in 2021.

## 3 Results

**Fig 1:**
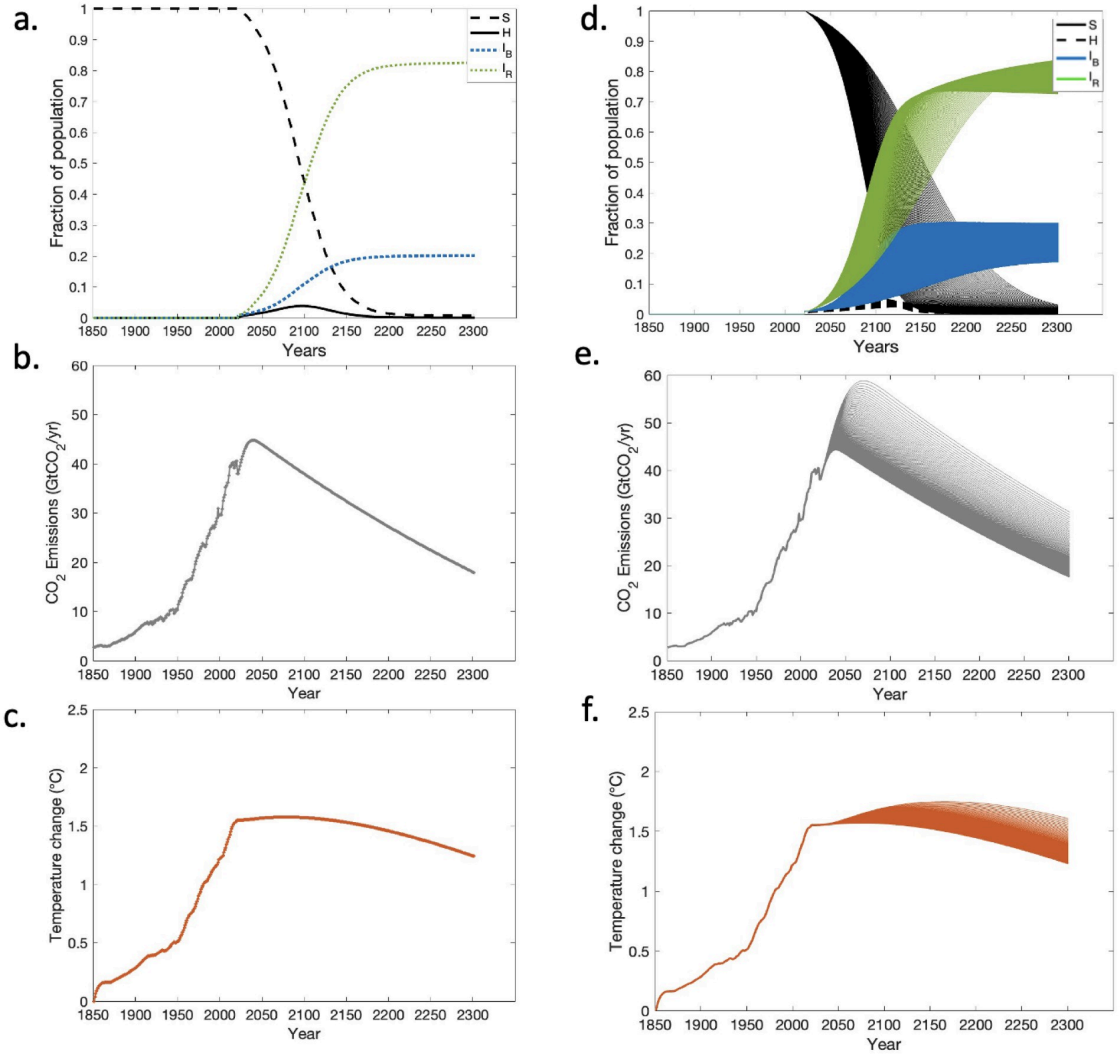
Dynamics of the coupled model for the baseline parameters. (a,d) Times series showing the rumor dynamics for baseline parameter values (b,e) Time series of emission scenario (GtCO_2_yr^-1^) and (c,f) temperature anomaly (°*C*) for baseline parameter values. (a,b,c) is the time series for the baseline parameter value and (d,e,f) is the time series plot generated using the accepted parameter values after applying ABC. Each of the multiple lines in (d,e,f) is a time series generated using each set of parameter values that were generated by ABC.

The model illustrates how social behavior, through rumor propagation, influences temperature levels. Initially, the whole population is susceptible to the rumors ($s_{0}=1,h_{0}=0,i_{b_{0}}=0,i_{r_{0}}=0$). A time series examination of the model indicates that a growing proportion of rejectors in the population causes lower emission levels depending on their emission-limiting strategies, as expected (Fig 1(a) and 1(b)). The transmission of multiple rumors and that a believer (rejector) believes (rejects) all the rumors that come up over time is considered. The result shows an optimistic case where 80% of rejectors are present in the population, with strong emission-limiting strategies to keep the temperature anomaly below 1.5°*C* (Fig 1(b)), which is considered to be an important climate change threshold [4].

Human actions predominantly affect climate through increased emissions, leading to the accumulation of greenhouse gases in the atmosphere, leading to severe and potentially irreversible changes. In this model, rumor propagation begins in 2021. The emissions and corresponding temperature anomalies before 2021 are computed using the anthropogenic emission data [56]. When individuals encounter rumors about climate change–such as climate change is not a serious problem or climate change is not real–they think about it and initially hesitate to decide whether to believe or reject it. The hesitating state can simply be considered as the state in which individuals are contemplating or checking facts before making their decision. Later, they decide whether to believe or not believe the rumors. The rejectors do not further spread the rumor, act like mitigators, and try to limit their emissions to reduce the impact of climate change, whereas the believers forward the rumor to more individuals, and the rumor spreads through the population.

Individuals stop believing the series of rumors when they experience a temperature change. As the proportion of rejectors increases in the population, the emission level slowly decreases, and so does the temperature. The temperature change is due to the cumulative amount of greenhouse gases in the atmosphere, especially CO_2_. The atmospheric CO_2_ does not reduce immediately but slowly dissipates, and the temperature decreases gradually as the CO_2_ level decreases. Even though the population starts to act immediately, it takes more time to see a change since it takes more time to dissipate the CO_2_ and even more time to see a drop in temperature.

To understand the influence of the emission limiting strategies considered by the rejectors in the population on the temperature anomaly, three scenarios are identified: a best-case scenario, which will be an ideal case where the temperature levels stay below the IPCC proposed levels [4], an average-case scenario, where the temperature increases little above the proposed level and a worst-case scenario, where the temperature increases far above the proposed level.

**Fig 2:**
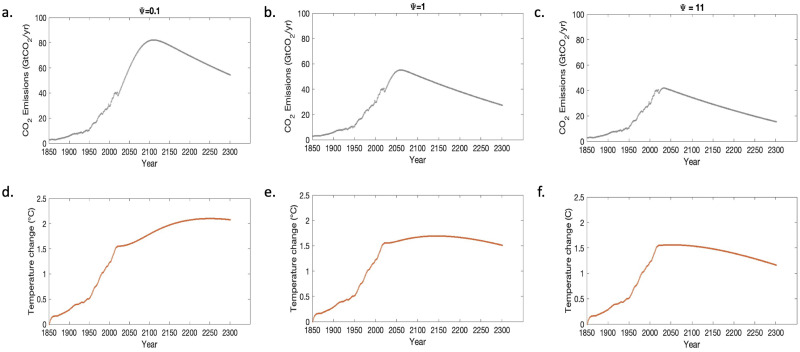
Emission scenarios for limiting global temperature change. (a) Emissions levels at Ψ = 0.1 (d) Temperature anomaly < 2.5°*C* at Ψ = 0.1 (b) Emission levels at Ψ = 1 (e) Temperature anomaly at 1.5°*C* (c)Emission levels when Ψ = 11 (f) Temperature anomaly at < 1.5°*C* with all other parameters at the baseline values.

**Best Case scenario**. An ideal case would be to limit the average global temperature change below 1.5°*C*. When the emission limiting constant Ψ is high (Ψ = 11), the temperature stays below the desired level and accomplishes the world’s climate goal (Fig 2(c) and 2(f)). The emissions decrease within a decade after the rumor propagation begins due to the strong mitigation strategies considered by the rejectors. Keeping the temperature rise below 1.5°*C* helps avoid irreversible changes like species extinction and adaptation problems. Our model suggests immediate mitigative actions are required to achieve our climate goals.

**Average Case scenario**. When the emission limiting constant Ψ takes a lower value indicating fewer mitigative actions (Ψ = 1), the emission increases for four decades before declining (Fig 2(b) and 2(e)). In this case, the temperature rises above 1.5°*C* and decrease slowly, leading to irreversible environmental changes. Our model predicts that four decadal difference in emission reduction lead to a 200-year delay in achieving our climate goal.

**Worst Case Scenario**. If the emission level does not decrease for another nine decades, the temperature does not decrease till 2300(Fig 2(a) and 2(d)). This situation arises due to poor or no mitigation steps taken by rejectors (Ψ = 0.1). The temperature increases above 2°*C* and does not decrease to a desired level, which could lead to unadaptable living conditions.

These three scenarios portray the importance of emission mitigating strategies. Even though the population consists of 80% rejectors, the efficiency of their emission-limiting strategies plays a vital role. This result shows that understanding that climate change is a major issue is not enough but to efficiently mitigate is also necessary.

### 3.1 Sensitivity analysis

The time series analysis gave an overall idea about how the model works but to further understand the influence of the model parameters, a sensitivity analysis is performed. The parameter planes generated by varying two model parameters simultaneously by retaining all other parameters at the baseline values help to understand how the simultaneous variation in two model parameters influences the model behavior at different time points. Time snapshots of parameter planes for emissions and temperature levels in 2021, 2070, and 2200 are considered.

**Fig 3:**
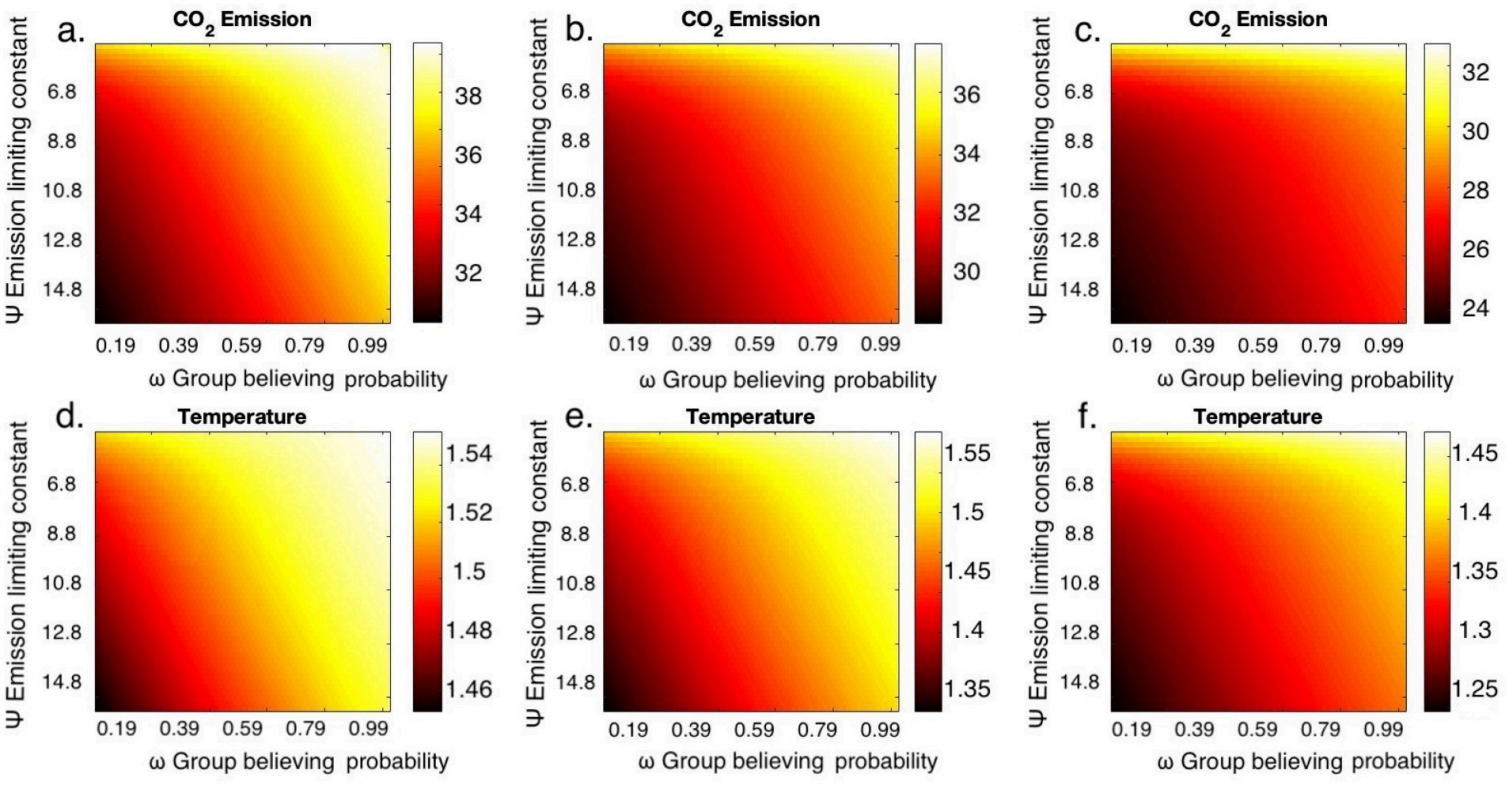
Temperature sensitivity is more with variation in emission limiting constant than group believing probabilities. Parameter planes of CO_2_ emissions (GtCO_2_yr^-1^) at (a) 2021, (b) 2070, and (c) 2200 and temperature anomaly (°*C*) at (d) 2021, (e) 2070 and (f) 2200 by varying group believing probability (*ω*) and emission limiting constant (Ψ).

**Fig 4:**
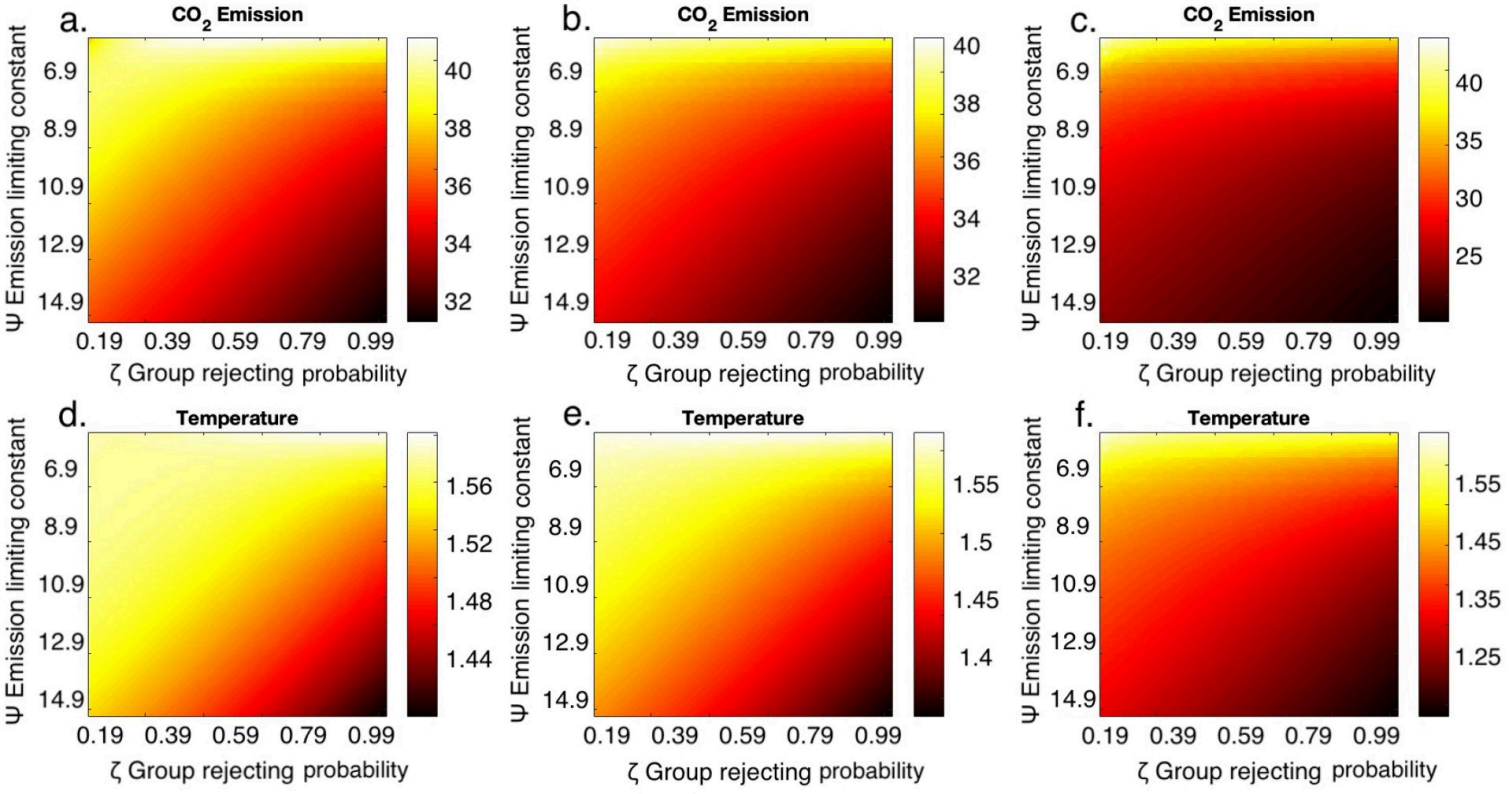
Global temperature anomaly is more sensitive to the emission-liming constant than group rejecting probabilities. (a-c) Parameter planes of CO_2_ emissions (GtCO_2_yr^-1^) at (a) 2021, (b) 2070, and (c) 2200 and (d-f) temperature anomaly (°*C*) at (d) 2021, (e) 2070 and (f) 2200 by varying group rejecting probability (*ζ*) and emission limiting constant (Ψ).

Emissions levels and temperature anomaly are more sensitive to changes in the emission-limiting value, Ψ, than the changes in believing and rejecting probabilities (Figs 3 and 4). This means that the strength with which rejectors limit their emissions through various mitigation strategies can significantly reduce emission and temperature levels, which reestablishes the result from Fig 2. This also indicates that efforts to mitigate climate change are as important as the number of individuals who support mitigation. In other words, having individuals who adopt mitigative measures is as important as having individuals who acknowledge the climate crisis. Simultaneously increasing the probability of rejection and decreasing the probability of believing leads to lower levels of emissions and temperature (S2 Fig). This was done to understand how the combination of both these probabilities would aid in reducing emissions and it was observed that both believing and rejecting probabilities contribute to the temperature anomaly equally. The emission limit constant (Ψ) has a greater influence on emission levels and temperature anomaly compared to the probabilities of believing (*ω*) and rejecting (*ζ*) (Figs 3 and 4) when the emission limiting strategies considered are lower. Decreasing the emission-limiting constant value after achieving the desired temperature goal can also cause an increase in temperature, which implies that reducing the efficiency of mitigation strategies once the target is achieved can take us to the initial scenario again. Hence it is important to maintain the efficiency in mitigating.

The emission levels and temperature anomaly decrease as the forgetting probability in believers increases and the forgetting probability in rejectors decreases (S3 Fig). As more rejectors forget about the rumors, they become susceptible again, according to the rumor propagation model, and hence the mitigation strategies decrease with a decrease in the rejector population. Whereas when more believers forget about the rumors, this leads to reduced emissions. The snapshots display that emission levels and the temperature anomaly are equally sensitive to both parameters.

The global temperature anomaly becomes less sensitive to the variation in believing or rejecting probabilities along with their corresponding forgetting probabilities as time passes (S4 and S5 Figs). Fewer parameter combinations lead to higher temperatures in 2200 than in 2070 and 2021, which implies that changing the believing (*ω*) or rejecting (*ζ*) probabilities and forgetting probabilities (*ρ*, *σ*) does not impact the temperature and emission levels after the rumor dynamics becomes stable. Also, the temperature anomaly is more sensitive to the forgetting probability in rejectors than the forgetting probabilities in believers (S4 and S5 Figs). High forgetting probabilities in rejectors lead to higher emissions and temperature levels. Maximum emissions are found at higher forgetting probabilities (in rejectors) and lower rejecting probabilities (S5 Fig). Rejectors forgetting the rumor has greater risks than the believers forgetting the rumor as rejectors act as mitigators and are the ones who adopt mitigative strategies to combat with climate change.

Univariate sensitivity analysis enables us to understand how sensitive the model is to a single parameter when it varies along its range by holding all other parameters at the baseline value. Only three points in the time series are considered to generate the univariate sensitivity plot: the peak value, the value in 2050, and the value in 2200.

Varying the total number of groups, *X* along its range (S1 Table) helps to understand the sensitivity of the model compartments concerning the parameter (S6 Fig). The baseline value for *X* is 40. A lower number of groups in the system leads to a sluggish rumor spreading. At lower *X* values, most of the population belongs in the susceptible compartment, and few are in the believer and rejector categories. The emission and temperature levels are also higher at lower *X* values than the higher ones, which implies that more groups assist in propagating the rumor quickly and assist in reducing the temperature. This portrays the importance of group propagation as shown in [49].

The univariate sensitivity plot attained by varying the average number of group members (*m*) displays that rumor propagation is quicker when the average number of group members is higher (S7 Fig). The baseline value for *m* for the model is 70. At lower *m* values, rumor propagation is considerably slow. It also leads to a higher temperature and CO_2_ emissions at lower *m* values which means that rumor propagation in groups with fewer members is less capable of spreading the rumor. Having more members in a group elevates the speed of the rumor propagation, making more individuals to decide at a time.

The univariate analysis for the parameter $\bar{c}$, which is the average number of contact for each individual, shows that higher $\bar{c}$ (baseline value 27) does not aid much in decreasing the temperature or the rumor propagation (S8 Fig) whereas lowering it slows down the rumor propagation leading to an elevated temperature level. Even though having more individual connections does not help in reducing the temperature, having fewer connections can certainly increase the temperature anomaly by making the information spread more localized. S9 Fig is the univariate analysis for the parameter $\bar{d}$, which is the average rate of transmitting messages per group. As the $\bar{d}$ value is decreased, the rumor propagation slows down, and the temperature shoots up. There is a considerable variation in the temperature anomaly as $\bar{d}$ decreases, implying that lowering the number of messages transmitted in the group can increase the temperature level, as it slows the rumor transmission among the population and making fewer individuals think about climate change. The parameter distribution for each model parameter is calculated using Approximate Bayesian Computation is in S10–S16 Figs.

**Fig 5:**
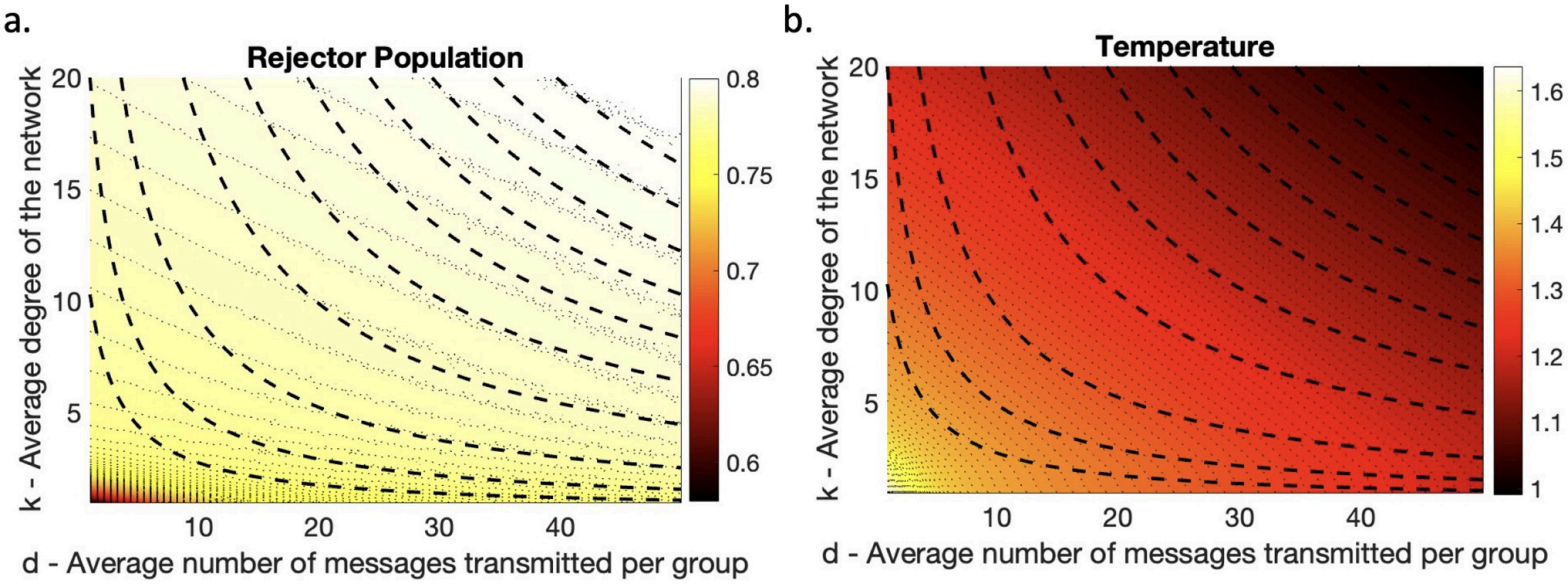
Global temperature anomaly is unaffected by low network degree at a high number of group messages. Parameter plane showing the changes in (a) rejector population and (b) temperature anomaly due to the variation in the average number of messages transmitted per group ($\bar{d}$) and the average degree of the network (*k*). The dashed lines show equal $\bar{d}*k$ values and the gray lines show equal size of rejector population and equal temperature anomaly.

The parameter plane by varying *k* and $\bar{d}$ illustrates that the temperature anomaly decreases as the average network degree and the messages transmitted per group increase (Fig 5). The hypothesis was that reducing the average degree of the network will not much affect the rumor propagation and temperature anomaly when the average number of messages transmitted in the groups is high. This hypothesis is tested using parameter planes by changing the parameters simultaneously, and the result supports our hypothesis. The dotted lines in Fig 5 represent equal products of the parameters (*d* * *k*). The constant product value (*d* * *k*), helps in analyzing how the rejector population and temperature anomaly vary, with changes in the parameter values.

The size of the rejector population determines the temperature anomaly, for instance, a higher rejector population leads to a lower temperature anomaly (Fig 5(a) and 5(b)). Higher variations in the size of the rejector population (and temperature anomaly) are observed when the average number of messages transmitted is low. This implies that both rejector population and temperature anomaly are more sensitive to the variations in the average number of messages transmitted per group than the network degree. In other words, the messaging rate in groups is more important than the number of individual connections in terms of determining the size of the rejector population (and thus in determining how much climate change is mitigated). As long as the frequency of messages about climate change in groups remains high, reduction in individual connections does not influence the temperature anomaly.

The interactions between rumor propagation parameters (group believing and rejecting probabilities) and the forcing due to doubling of CO_2_ (Wm^-2^) shows that temperature anomaly is more sensitive to changes in forcing than the changes in the believing or rejecting probability (S17 Fig). The forcing due to doubling of CO_2_ (F2x), causes an increase in the temperature anomaly even at lower believing probability (higher rejecting probability). Being a an environmental variable, forcing has its own upper hand in influencing climate change compared to human actions but human actions are the reason for these changes is also an underlying fact.

The tornado diagram is a sensitivity analysis tool that helps examine the model parameters’ relative priority. The sensitivity of the temperature anomaly (in 2200) demonstrates the relative sensitivity of each parameter. S18 Fig is the tornado plot showing the temperature anomaly sensitivity when the model parameters vary individually at 10%. The analysis confirms the results from S18 Fig that temperature change is most sensitive to the emission limiting constant Ψ (S18 Fig). The temperature is more sensitive to the group parameters than the individual parameters. The hypothesis that temperature change is more sensitive to forgetting probability in rejectors is also true in the case of 10% change in the parameters even though there are no visible changes because of the extremely low baseline value considered. The tornado plot confirms the results of the previous sensitivity analysis and helps to understand the relative influence for each parameter.

## 4 Discussion

A rumor propagation model was linked with an Earth System model to understand how the spread of climate rumors responds to climate change, and how resulting behavioral change can feedback on climate. Differing from the existing literature, this model focuses on how rumors about climate change can influence the climate system itself, using methods from compartmental modeling and Earth System Modeling [59–65]. The study has shown that the mitigation strategies by rumor rejectors to limit emissions are vital in reducing emission levels and temperature anomalies. Acknowledging that climate change is a major issue is not enough but working efficiently towards mitigation and adopting efficient mitigation strategies are vital in controlling the effects of climate change. The parameter planes and tornado plots suggest that temperature anomaly is most sensitive to these mitigation strategies by the rejectors. The mitigation plan is the crucial link that connects human behavior and climate change in our model. Our results support that human mitigation strategies help in keeping climate change under control by avoiding undesirable and uninhabitable situations in the future, similar to the suggestions from various studies [29–32, 66]. The extent of climate change we will experience in the future depends on the strategies we adopt today and how soon we start acting according to the strategies adopted. The more time we take to reduce our emissions, the higher the average global temperature anomaly will be. The coupled rumor-climate model suggests that misinformation via rumors is a significant issue. The effort the rejectors make to mitigate climate change is vital in controlling the time taken for decreasing the emission level, and temperature changes.

Our simple rumor-climate model shows that the temperature anomaly is more sensitive to mitigating strategies than believing or not believing the climate rumors. Surprisingly, the temperature becomes less sensitive to the variation in rumor propagation parameters as time passes. Our results show that larger groups assist in rapid rumor propagation and hence in temperature deviations. The univariate sensitivity plots infer that having larger groups with more members is more efficient in propagating rumors and reducing the temperature than having more groups with fewer members. In other words, the decision of a group with more members has more influence on the temperature levels than smaller groups. The coupled model also portrays that group propagation is faster than individual propagation and hence has more influence on the temperature anomaly. Decreasing the connectivity of individuals does not influence the number of rejectors in the population when a high volume of rumors is sent in groups. The average rate of messages sent in groups produces more variation in the temperature levels, and temperature is more sensitive to group propagation parameters than individual propagation parameters. Our model deals with arbitrary decision-making in the rumor propagation model by considering that the whole population either believes or rejects the rumors. In contrast, decision-making might be influenced by the individual’s cultural, political, educational, or social views, which is not covered in this model. The spread of rumors about climate change can be controlled using various methods such as inoculations through educational approaches and social norms [67]. Proper climate education is necessary for individuals to distinguish between rumors and facts about climate change and take the right decisions by considering mitigation and adaptation strategies.

Several models deal with climate change, but models involving human influence on climate change are rare. The recent development of coupled social-climate models have included social and economic factors [27, 33, 37], but ours is the first social-climate model to include rumor propagation, to our knowledge. Social networks act as an essential medium of information circulation, therefore it is crucial to understand how rumor propagation in a heterogeneous social network can influence individuals’ opinions on climate change. According to our model, social networks have a vital role in influencing the climate system. Coupled models can be beneficial in including dynamic social processes in climate models. Including these models in climate change research can provide different perspectives and predictions regarding climate change. Coupled models can also help in policy-making by providing novel emission scenarios based on social mechanisms that are known to be important. Also, according to our model, using individuals in social networks to become aware of climate change issues could be beneficial to reduce emissions by increasing the number of mitigators in society. Social networks act as an important way of spreading information regarding climate change and inviting more individuals to act at the right time.

## Supporting information

S1 Fig Flow chart with steps of conducting the study. (TIFF)

S2 Fig Temperature change is sensitive to both believing and rejecting probability in groups. (a-c)Parameter planes of CO_2_ emissions (GtCO_2_ yr^-1^) at (a) 2021, (b) 2070, and (c) 2200 and (d-f) temperature changes (°*C*) at (d) 2021, (e) 2070 and (f) 2200 by varying group believing probability (*ω*) and group rejecting probability (*ζ*).
(TIF)

S3 Fig Sensitivity of temperature change to forgetting probability in both believers and rejectors. (a-c)Parameter planes of CO_2_ emissions (GtCO_2_ yr^-1^) at (a) 2021, (b) 2070, and (c) 2200 and (d-f) temperature changes (°*C*) at (d) 2021, (e) 2070 and (f) 2200 by varying group forgetting probability in believers (*ρ*) and group forgetting probability in rejectors (*σ*).
(TIF)

S4 Fig Effect of variations in a group believing probability and forgetting probability on temperature levels. (a-c)Parameter planes of CO_2_ emissions (GtCO_2_ yr^-1^) at (a) 2021, (b) 2070, and (c) 2200 and (d-f) temperature changes (°*C*) at (d) 2021, (e) 2070 and (f) 2200 by varying group believing probability (*ω*) and group forgetting probability in believers (*ρ*).
(TIF)

S5 Fig Effect of variations in a group rejecting probability and forgetting probability on temperature levels. (a-c)Parameter planes of CO_2_ emissions (GtCO_2_ yr^-1^) at (a) 2021, (b) 2070, and (c) 2200 and (d-f) temperature changes (°*C*) at (d) 2021, (e) 2070 and (f) 2200 by varying the group rejecting probability (*ζ*) and group forgetting probability in rejectors (*σ*).
(TIF)

S6 Fig Changes in model dynamics with variation in total number of groups. Univariate sensitivity of (a) CO_2_ emissions, (b) temperature, (c) susceptible, (d) hesitator, (e) believer, and (f) rejector population by varying the parameter *X*, the total number of groups.
(TIF)

S7 Fig Changes in model dynamics with variation in average number of group members. Univariate sensitivity of (a) CO_2_ emissions, (b) temperature, (c) susceptible, (d) hesitator, (e) believer, and (f) rejector population by varying the parameter *m*, the average number of group members.
(TIF)

S8 FigChanges in model dynamics with variation in average number of contact for each individual.Univariate sensitivity of (a) CO_2_ emissions, (b) temperature, (c) susceptible, (d) hesitator, (e) believer, and (f) rejector population by varying the parameter $\bar{c}$, the average number of contact for each individual.(TIF)

S9 FigChanges in model dynamics with variation in the average rate of transmitting messages per group.Univariate sensitivity of (a) CO_2_ emissions, (b) temperature, (c) susceptible, (d) hesitator, (e) believer, and (f) rejector population by varying the parameter $\bar{d}$- the average rate of transmitting messages per group.(TIF)

S10 Fig Parameter values distribution of (a) *β* Individual hesitating probability and (b) *η* Individual believing probability generated using ABC. (TIF)

S11 Fig Parameter values distribution of (c) *α* Believer forgetting probability and (d) *γ* rejector forgetting probability generated using ABC. (TIF)

S12 Fig Parameter values distribution of (e) *ς* Group hesitating probability and (f) *ω* Group believing probability generated using ABC. (TIF)

S13 Fig Parameter values distribution of (g) *ρ* Group believer forgetting probability and (h) *σ* Group rejector forgetting probability generated using ABC. (TIF)

S14 FigParameter values distribution of (i) $\bar{c}$ Average rate of contact per individual and (j) $\bar{d}$ Average rate of transmitting message per group generated using ABC.(TIF)

S15 Fig Parameter values distribution of (k) *k* Average degree of the network and (l) Ψ Emission limiting constant generated using ABC. (TIF)

S16 Fig Parameter values distribution of (m) *X* Total number of groups and (n) *m* Average number of group members generated using ABC. (TIF)

S17 Fig Interaction between forcing due to CO_2_ doubling and rumor parameters. (TIF)

S18 Fig Sensitivity of temperature anomaly at 10% change in parameter values. Tornado plot showing the sensitivity of temperature change when parameters are varied individually, with a 10% increase and decrease from the baseline values. The red color represents an increase in the parameter value and the blue color represents a decrease in the parameter value, from the baseline value.
(TIF)

S1 Table Table describing the model parameters and values. (PDF)

S1 Text Stability analysis for the coupled model. (PDF)

## References

[1] Petritan Any Mary., Schwenke Mirela Beloiu. Forest Functioning under Climate Warming and Future Perspectives on Forest Disturbances. Forests, (2023).

[2] Dollinger Christina., Rammer Werner., Seidl Rupert. Climate change accelerates ecosystem restoration in the mountain forests of Central Europe. Journal of Applied Ecology, (2023). doi: 10.1111/1365-2664.14520

[3] Forzieri Giovanni., Dakos Vasilis., McDowell Nate G., Ramdane Alkama., Cescatti Alessandro. Emerging signals of declining forest resilience under climate change. Visual education, (2022). doi: 10.1038/s41586-022-04959-9 35831499 PMC9385496

[4] Masson-Delmotte V, Zhai P, Pörtner H-O, Roberts D, Skea J, Shukla PR, et al. Global warming of 1.5°C. An IPCC Special Report on the impacts of global warming of 1.5°C. 2018.

[5] Echendu Adaku Jane., Okafor Henry Favour., Iyiola Olayinka. Air Pollution, Climate Change and Ecosystem Health in the Niger Delta. Advances in the Social Sciences, (2022).

[6] Kumar Ashok., Omidvarborna Hamid., Shandilya Kaushik K. Air Pollution and Climate Change: Relationship Between Air Quality and Climate Change. (2018).

[7] Strefler Jessica., Luderer Gunnar., Kriegler Elmar., Meinshausen Malte. Can air pollutant controls change global warming. Environmental Science and Policy, (2014). doi: 10.1016/j.envsci.2014.04.009

[8] Hassan WH, Nile BK, Mahdi K, Wesseling J, Ritsema C. A feasibility assessment of potential artificial recharge for increasing agricultural areas in the kerbala desert in iraq using numerical groundwater modeling. Water. 2021 Nov 10;13(22):3167. doi: 10.3390/w13223167

[9] Hassan WH, Hussein HH, Nile BK. The effect of climate change on groundwater recharge in unconfined aquifers in the western desert of Iraq. Groundwater for Sustainable Development. 2022 Feb 1;16:100700. doi: 10.1016/j.gsd.2021.100700

[10] Hassan WH. Climate change projections of maximum temperatures for southwest Iraq using statistical downscaling. Climate Research. 2021 May 6;83:187–200. doi: 10.3354/cr01647

[11] Masson-Delmotte V, Zhai P, Pörtner H-O, Roberts D, Skea J, Shukla PR, et al. Global warming of 1.5°C. An IPCC Special Report on the impacts of global warming of 1.5°C. 2019.

[12] Capstick S, Lorenzoni I, Corner A, Whitmarsh L. Prospects for radical emissions reduction through behavior and lifestyle change. Carbon Manage. 2014;5(4):429–45. doi: 10.1080/17583004.2015.1020011

[13] Green R, Milner J, Dangour AD, Haines A, Chalabi Z, Markandya A, et al. The potential to reduce greenhouse gas emissions in the UK through healthy and realistic dietary change. Clim Change. 2015;129:253–65. doi: 10.1007/s10584-015-1329-y

[14] Dietz T, Gardner GT, Gilligan J, Stern PC, Vandenbergh MP. Household actions can provide a behavioral wedge to rapidly reduce US carbon emissions. Proc Natl Acad Sci USA. 2009;106(44):18452–6. doi: 10.1073/pnas.0908738106 19858494 PMC2767367

[15] Manabe S, Bryan K. Climate calculations with a combined ocean-atmosphere model. J Atmos Sci. 1969;26(4):786–9. doi: 10.1175/1520-0469(1969)026<0786:CCWACO>2.0.CO;2

[16] Sellers PJ, Mintz YC, Sud YC, Dalcher A. A simple biosphere model (SiB) for use within general circulation models. J Atmos Sci. 1986;43(6):505–31. doi: 10.1175/1520-0469(1986)043<0505:ASBMFU>2.0.CO;2

[17] Wilson MF, Henderson-Sellers A, Dickinson RE, Kennedy PJ. Sensitivity of the Biosphere–Atmosphere Transfer Scheme (BATS) to the inclusion of variable soil characteristics. J Appl Meteorol Climatol. 1987;26(3):341–62. doi: 10.1175/1520-0450(1987)026<0341:SOTBTS>2.0.CO;2

[18] Bonan GB, Doney SC. Climate, ecosystems, and planetary futures: The challenge to predict life in Earth system models. Science. 2018;359(6375):eaam8328. doi: 10.1126/science.aam8328 29420265

[19] Plattner GK, Joos F, Stocker TF, Marchal O. Feedback mechanisms and sensitivities of ocean carbon uptake under global warming. Tellus B: Chem Phys Meteorol. 2001;53(5):564–92. doi: 10.1034/j.1600-0889.2001.530504.x

[20] Field CB, Lobell DB, Peters HA, Chiariello NR. Feedbacks of terrestrial ecosystems to climate change. Annu Rev Environ Resour. 2007;32:1–29. doi: 10.1146/annurev.energy.32.053006.141119

[21] Baird T, Jacob A, Rosenstock T, Schindler S. Evaluating the impact of climate change adaptation on ecosystem services in the Lower Mekong Basin. Environ Sci Policy. 2014;44:306–16.

[22] Howe P, Markowitz E, Lee T, Kovacs P. Climate change: perceptions and behavioral responses. Nat Clim Chang. 2013;3(5):393–8.

[23] Clayton S, Manning C, Krygsman K, Speiser M. Mental health and well-being in the context of climate change. Clim Change. 2015;129(3):385–98.

[24] Spence A, Poortinga W, Butler C, Pidgeon N. Perceptions of climate change and willingness to change behavior. Global Environ Chang. 2011;21(1):5–13.

[25] Osbahr H, Davies M, Stringer LC. Climate change adaptation and livelihoods in the semi-arid zones of Zimbabwe. Appl Geogr. 2011;31(4):1176–84.

[26] Galvani AP, Bauch CT, Anand M, Singer BH, Levin SA. Human–environment interactions in population and ecosystem health. Proc Natl Acad Sci USA. 2016;113(51):14502–6. doi: 10.1073/pnas.1618138113 27956616 PMC5187669

[27] Bury TM, Bauch CT, Anand M. Charting pathways to climate change mitigation in a coupled socio-climate model. PLoS Comput Biol. 2019;15(6):e1007000. doi: 10.1371/journal.pcbi.1007000 31170149 PMC6553685

[28] Davis-Sowers R. Under the Influence: Putting Peer Pressure to Work. Int Soc Sci Rev. 2020;96(2):1.

[29] Leila N, Kiesewetter G, Wagner F, Schöpp W, Filatova T, Voinov A, et al. Assessing the macroeconomic impacts of individual behavioral changes on carbon emissions. Clim Change. 2019.

[30] Wynes S, Nicholas KA, Zhao J, Donner SD. Measuring what works: quantifying greenhouse gas emission reductions of behavioural interventions to reduce driving, meat consumption, and household energy use. Environ Res Lett. 2018. doi: 10.1088/1748-9326/aae5d7

[31] Khanna T, Baiocchi G, Callaghan M, Creutzig F, Guias H, Haddaway NR, et al. A multi-country meta-analysis on the role of behavioural change in reducing energy consumption and CO2 emissions in residential buildings. Nat Energy. 2021. doi: 10.1038/s41560-021-00866-x

[32] van de Ven DJ, González-Eguino M, Arto I. The potential of behavioural change for climate change mitigation: a case study for the European Union. Mitig Adapt Strateg Glob Change. 2018;23(6):853–886. doi: 10.1007/s11027-017-9763-y

[33] Weber EU. What shapes perceptions of climate change? WIREs Clim Chang. 2010;1(3):332–42. doi: 10.1002/wcc.41

[34] Hargreaves T. Practice-ing behaviour change: Applying social practice theory to pro-environmental behaviour change. J Consum Cult. 2011;11(1):79–99. doi: 10.1177/1469540510390500

[35] Schlüter M, Baeza A, Dressler G, Frank K, Groeneveld J, Jager WA, et al. A framework for mapping and comparing behavioural theories in models of social-ecological systems. Ecol Econ. 2017;131:21–35. doi: 10.1016/j.ecolecon.2016.08.008

[36] Taylor KE, Stouffer RJ, Meehl GA. An overview of CMIP5 and the experiment design. Bull Am Meteorol Soc. 2012;93(4):485–98. doi: 10.1175/BAMS-D-11-00094.1

[37] Menard J, Bury TM, Bauch CT, Anand M. When conflicts get heated, so does the planet: coupled social-climate dynamics under inequality. Proc R Soc B. 2021;288(1959):20211357. doi: 10.1098/rspb.2021.1357 34521252 PMC8441127

[38] Thornton PE, Calvin K, Jones AD, Di Vittorio AV, Bond-Lamberty B, Chini L, Shi X, et al. Biospheric feedback effects in a synchronously coupled model of human and Earth systems. Nat Clim Chang. 2017;7(7):496–500. doi: 10.1038/nclimate3310

[39] Clarke L, Eom J, Marten EH, Horowitz R, Kyle P, Link R, et al. Effects of long-term climate change on global building energy expenditures. Energy Econ. 2018;72:667–77. doi: 10.1016/j.eneco.2018.01.003

[40] Nordhaus W. Evolution of modeling of the economics of global warming: changes in the DICE model, 1992-2017. Clim Chang. 2018;148(4):623–40. doi: 10.1007/s10584-018-2218-y

[41] Nordhaus W. Climate change: The ultimate challenge for economics. Am Econ Rev. 2019;109(6):1991–2014. doi: 10.1257/aer.109.6.1991

[42] Hoffman AJ. Climate change as a cultural and behavioral issue: Addressing barriers and implementing solutions. Organ Dyn. 2010;39(4):295–305. doi: 10.1016/j.orgdyn.2010.07.005

[43] McCright AM, Dunlap RE, Xiao C. Increasing influence of party identification on perceived scientific agreement and support for government action on climate change in the United States, 2006-12. Weather Clim Soc. 2014;6(2):194–201. doi: 10.1175/WCAS-D-13-00058.1

[44] Wilkins A. Machine-learning ensembled CMIP6 projection reveals socio-economic pathways will aggravate global warming and precipitation extremes. Hydrol Earth Syst Sci. 2022.

[45] Wu H. SCWIR: A rumor propagation model considering user waiting behavior. 2024.

[46] Huo L, Dong Y, Lin T. Rumor spreading model with a focus on educational impact and optimal control. Chinese Phys B. 2021.

[47] Li D. Rumor spreading model with a focus on educational impact and optimal control. 2023.

[48] Daley DJ, Kendall DG. Epidemics and rumours. Nature. 1964;204:1118. doi: 10.1038/2041118a0 14243408

[49] Myilsamy K, Kumar MS, Kumar AS. Optimal control of a rumor model with group propagation over complex networks. Int J Mod Phys C. 2021;32(03):2150035. doi: 10.1142/S0129183121500352

[50] Whitmarsh L. Behavior and climate change: The role of social norms. In: Climate Change and Society: Approaches to Understanding and Influencing Human Behavior. Routledge; 2009. p. 66–87.

[51] Mazid MA, Zarnaz Z. Climate change myths detection using dynamically weighted ensemble based stance classifier. 2022.

[52] Meddeb P, Ruseti S, Dascalu M, Terian S, Travadel S. Counteracting French fake news on climate change using language models. Sustainability. 2022;14(18):11724. doi: 10.3390/su141811724

[53] Taddicken M, Wolff LJ. Climate change-related counter-attitudinal fake news exposure and its effects on search and selection behavior. 2023. doi: 10.1055/s-0043-1761499 36750219 PMC10462433

[54] Rosa K. Influence of media framing on public perception of climate change. J Commun. 2024.

[55] Millar RJ, Nicholls ZR, Friedlingstein P, Allen MR. A modified impulse-response representation of the global near-surface air temperature and atmospheric concentration response to carbon dioxide emissions. Atmos Chem Phys. 2017;17(11):7213–7228. doi: 10.5194/acp-17-7213-2017

[56] Friedlingstein P, O’Sullivan M, Jones MW, Andrew RM, Hauck J, Olsen A, et al. Global carbon budget 2020. Earth Syst Sci Data. 2020;12(4):3269–3340. doi: 10.5194/essd-12-3269-2020

[57] Minter A, Retkute R. Approximate Bayesian Computation for infectious disease modelling. Epidemics. 2019;29:100368. doi: 10.1016/j.epidem.2019.100368 31563466

[58] Sunnaaker M, Busetto AG, Numminen E, Corander J, Foll M, Dessimoz C. Approximate Bayesian computation. PLoS Comput Biol. 2013;9(1):e1002803. doi: 10.1371/journal.pcbi.1002803 23341757 PMC3547661

[59] Beckage B, Gross LJ, Lacasse K, Carr E, Metcalf SS, Winter JM, et al. Linking models of human behaviour and climate alters projected climate change. Nat Clim Chang. 2018;8(1):79–84. doi: 10.1038/s41558-017-0031-7

[60] Beckage B, Moore FC, Lacasse K. Incorporating human behaviour into Earth system modelling. Nat Hum Behav. 2022;1–10. 36385182 10.1038/s41562-022-01478-5

[61] Wen S, Haghighi MS, Chen C, Xiang Y, Zhou W, Jia W. A sword with two edges: Propagation studies on both positive and negative information in online social networks. IEEE Trans Comput. 2014;64(3):640–53. doi: 10.1109/TC.2013.2295802

[62] Roets A, et al. ‘Fake news’: Incorrect, but hard to correct. The role of cognitive ability on the impact of false information on social impressions. Intelligence. 2017;65:107–10. doi: 10.1016/j.intell.2017.10.005

[63] Sahafizadeh E, Ladani BT. The impact of group propagation on rumor spreading in mobile social networks. Physica A: Stat Mech Appl. 2018;506:412–23. doi: 10.1016/j.physa.2018.04.038

[64] Youssef M, Scoglio C. An individual-based approach to SIR epidemics in contact networks. J Theor Biol. 2011;275(1):112–22. 21663750 10.1016/j.jtbi.2011.05.029

[65] Kessler JA, McGowan R, Paulson E. Exploring nonlinear dynamical systems for modeling the impact of climate policy on economic growth. J Econ Dyn Control. 2022;139:104459.

[66] Houghton JT. Global warming: The complete briefing. Cambridge University Press; 1997.

[67] Cook J. Understanding and countering misinformation about climate change. Information Science Reference/IGI Global; 2019.

