# Supplementary figures and images for: Climate-denying rumor propagation in a coupled socio-climate model: Impact on average global temperature
Source: PLoS One. 2025 Jan 16;20(1):e0317338. doi: 10.1371/journal.pone.0317338 (PMC11737659; doi:10.1371/journal.pone.0317338)

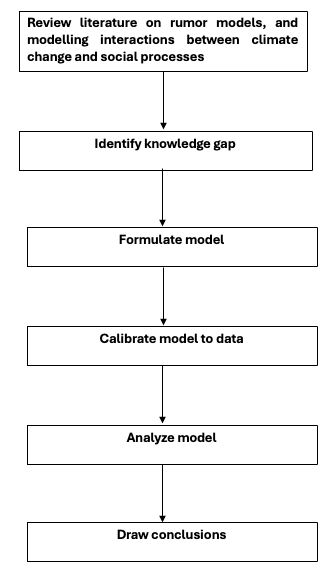

Supplement: S1 Fig — (TIFF) [file pone.0317338.s001.tiff]

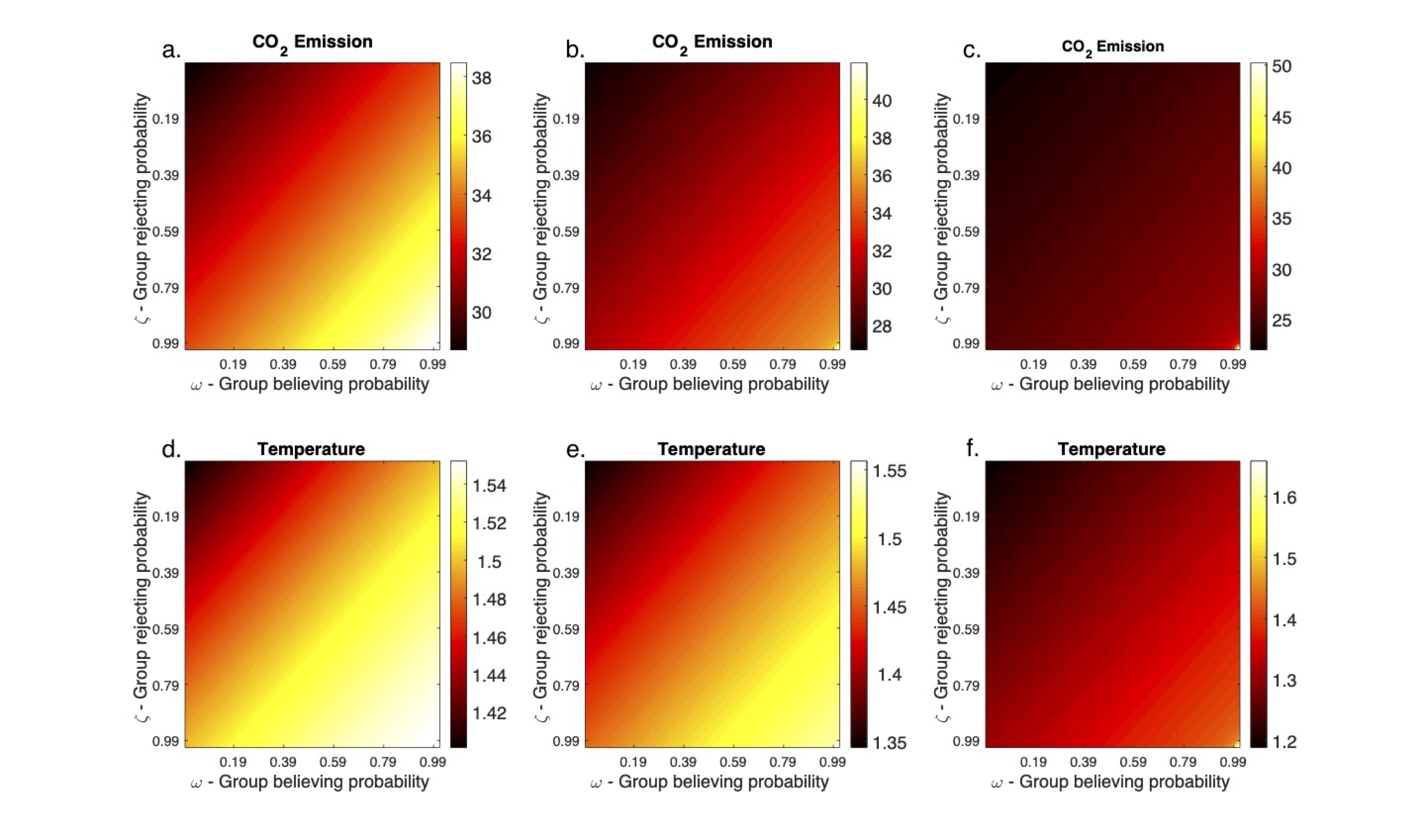

Supplement: S2 Fig — (a-c)Parameter planes of CO2 emissions (GtCO2 yr-1) at (a) 2021, (b) 2070, and (c) 2200 and (d-f) temperature changes (°C) at (d) 2021, (e) 2070 and (f) 2200 by varying group believing probability (ω) and group rejecting probability (ζ). (TIF) [file pone.0317338.s002.tif]

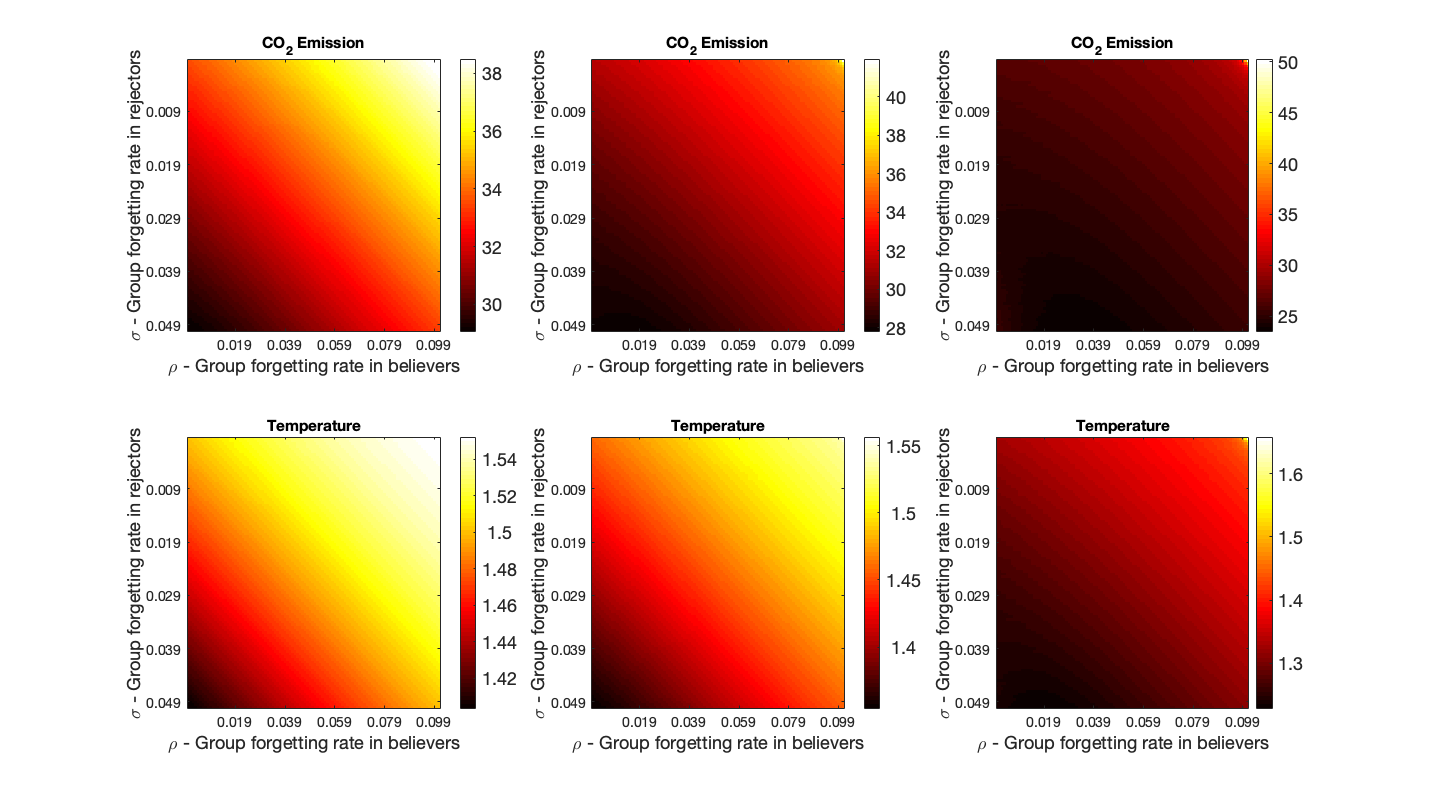

Supplement: S3 Fig — (a-c)Parameter planes of CO2 emissions (GtCO2 yr-1) at (a) 2021, (b) 2070, and (c) 2200 and (d-f) temperature changes (°C) at (d) 2021, (e) 2070 and (f) 2200 by varying group forgetting probability in believers (ρ) and group forgetting probability in rejectors (σ). (TIF) [file pone.0317338.s003.tif]

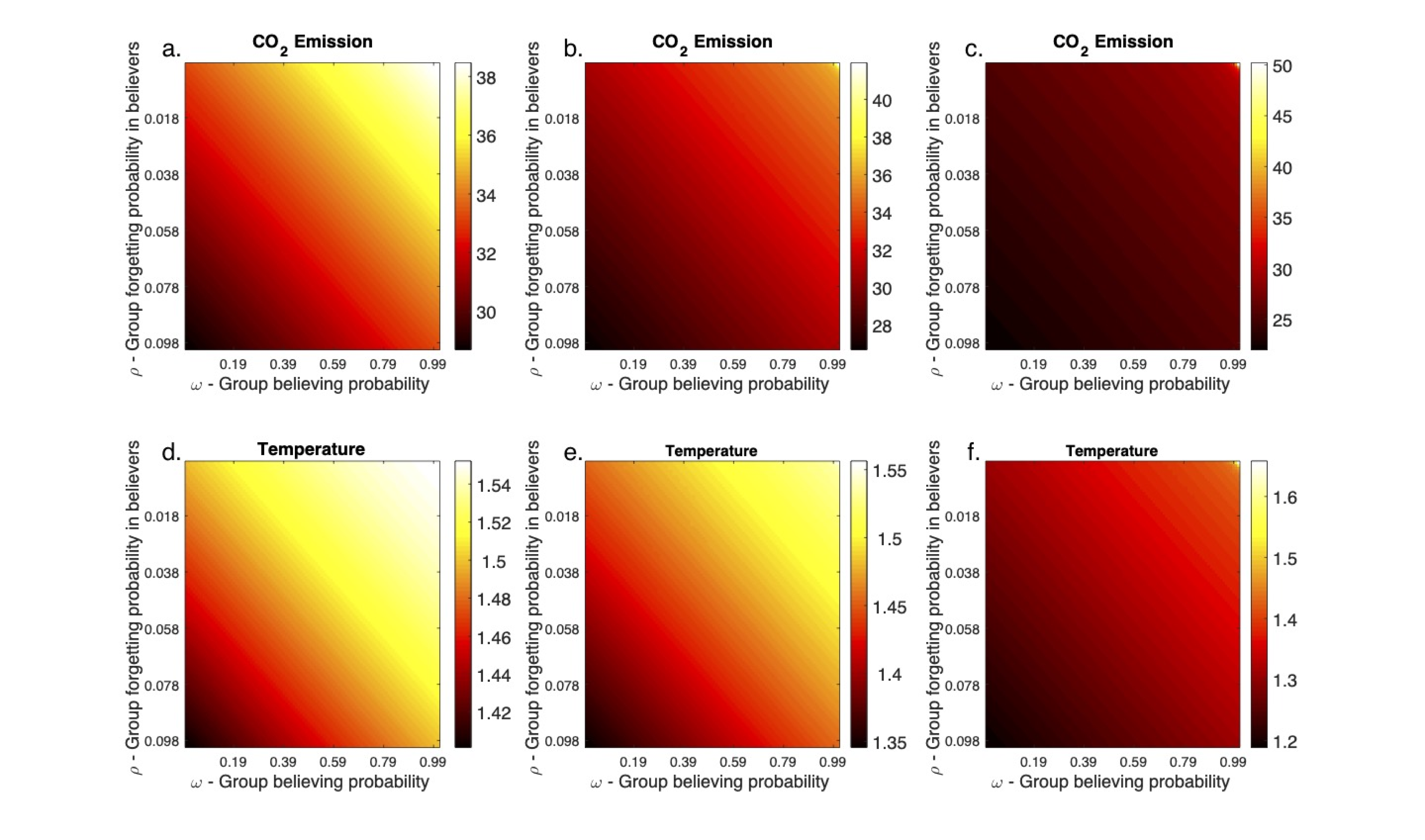

Supplement: S4 Fig — (a-c)Parameter planes of CO2 emissions (GtCO2 yr-1) at (a) 2021, (b) 2070, and (c) 2200 and (d-f) temperature changes (°C) at (d) 2021, (e) 2070 and (f) 2200 by varying group believing probability (ω) and group forgetting probability in believers (ρ). (TIF) [file pone.0317338.s004.tif]

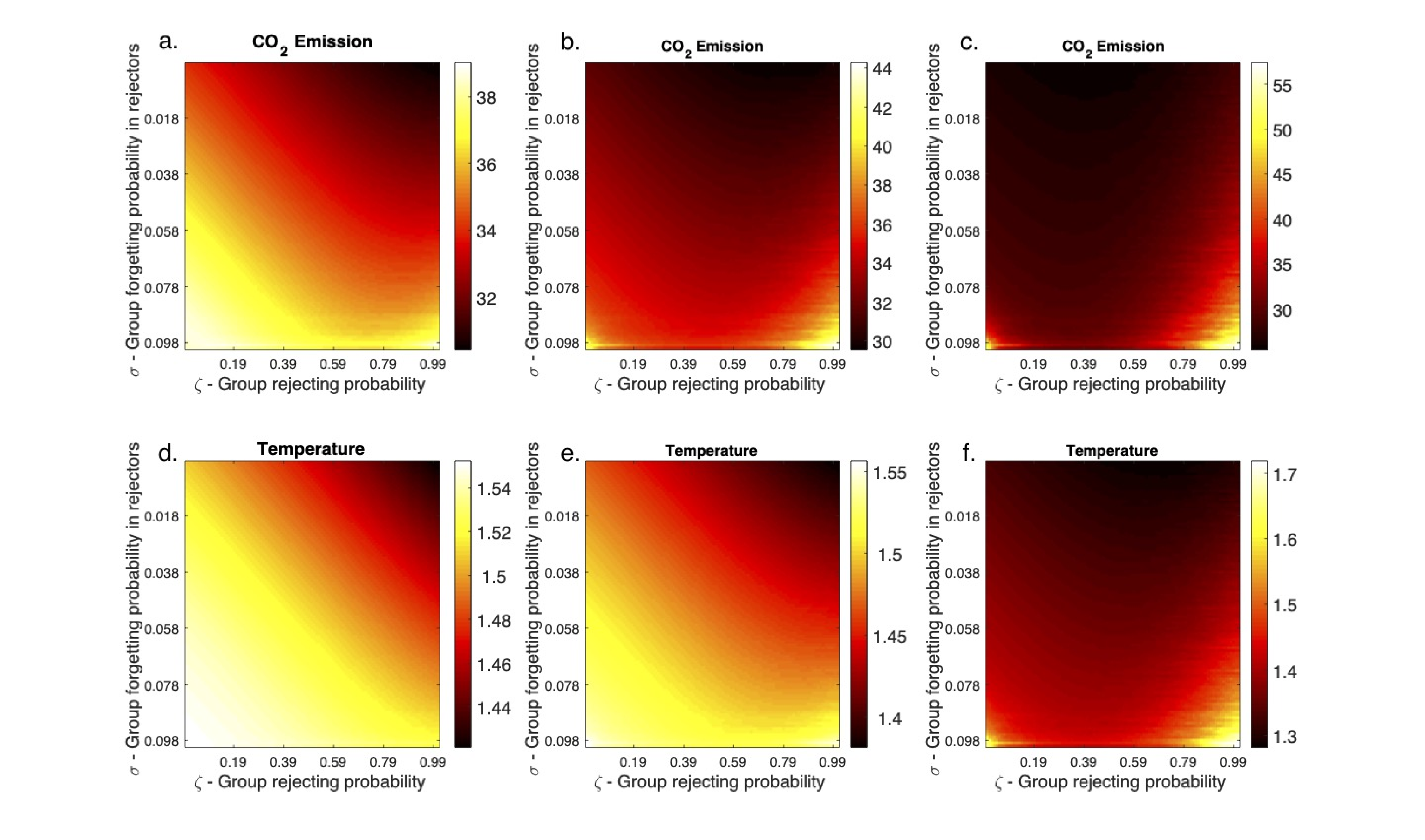

Supplement: S5 Fig — (a-c)Parameter planes of CO2 emissions (GtCO2 yr-1) at (a) 2021, (b) 2070, and (c) 2200 and (d-f) temperature changes (°C) at (d) 2021, (e) 2070 and (f) 2200 by varying the group rejecting probability (ζ) and group forgetting probability in rejectors (σ). (TIF) [file pone.0317338.s005.tif]

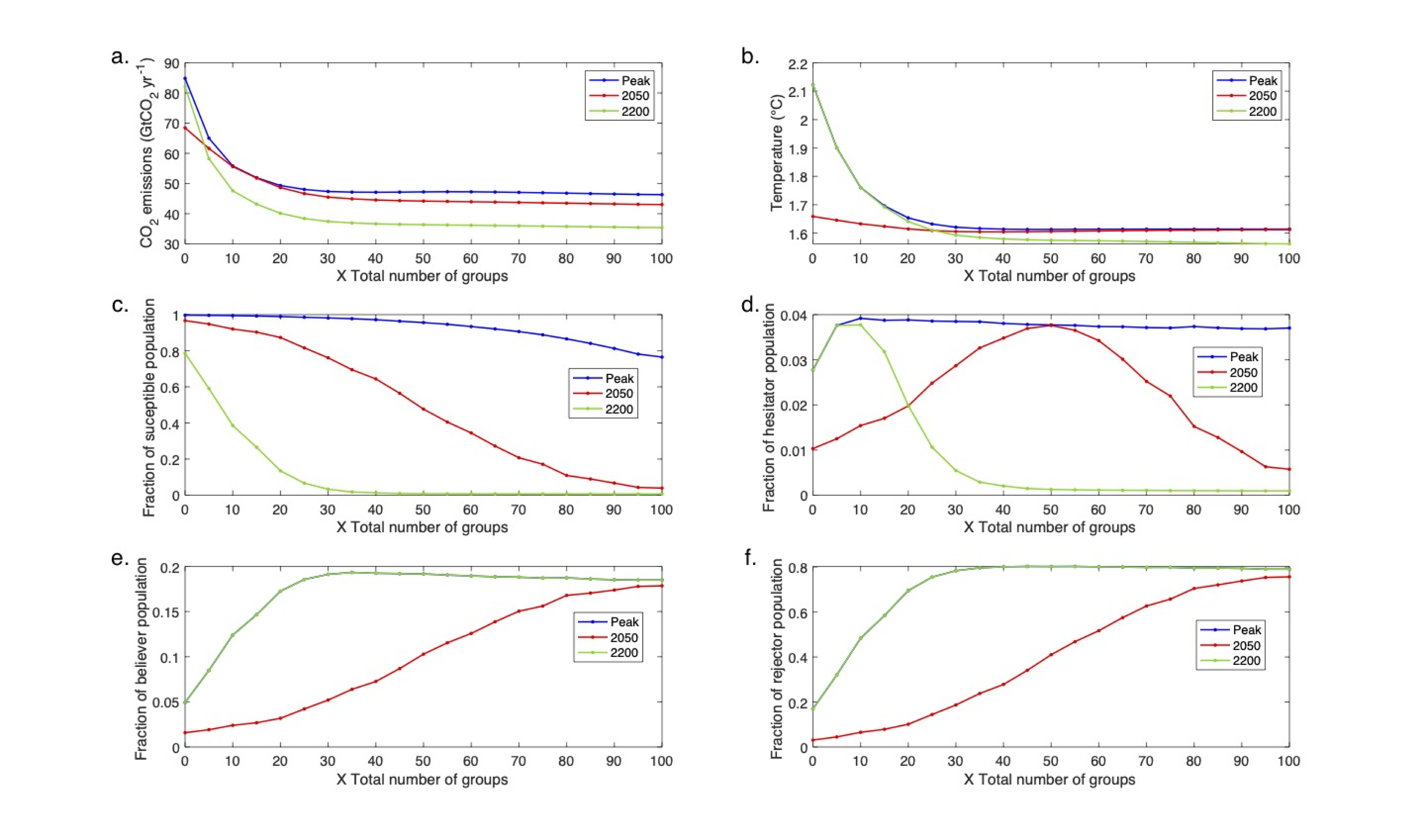

Supplement: S6 Fig — Univariate sensitivity of (a) CO2 emissions, (b) temperature, (c) susceptible, (d) hesitator, (e) believer, and (f) rejector population by varying the parameter X, the total number of groups. (TIF) [file pone.0317338.s006.tif]

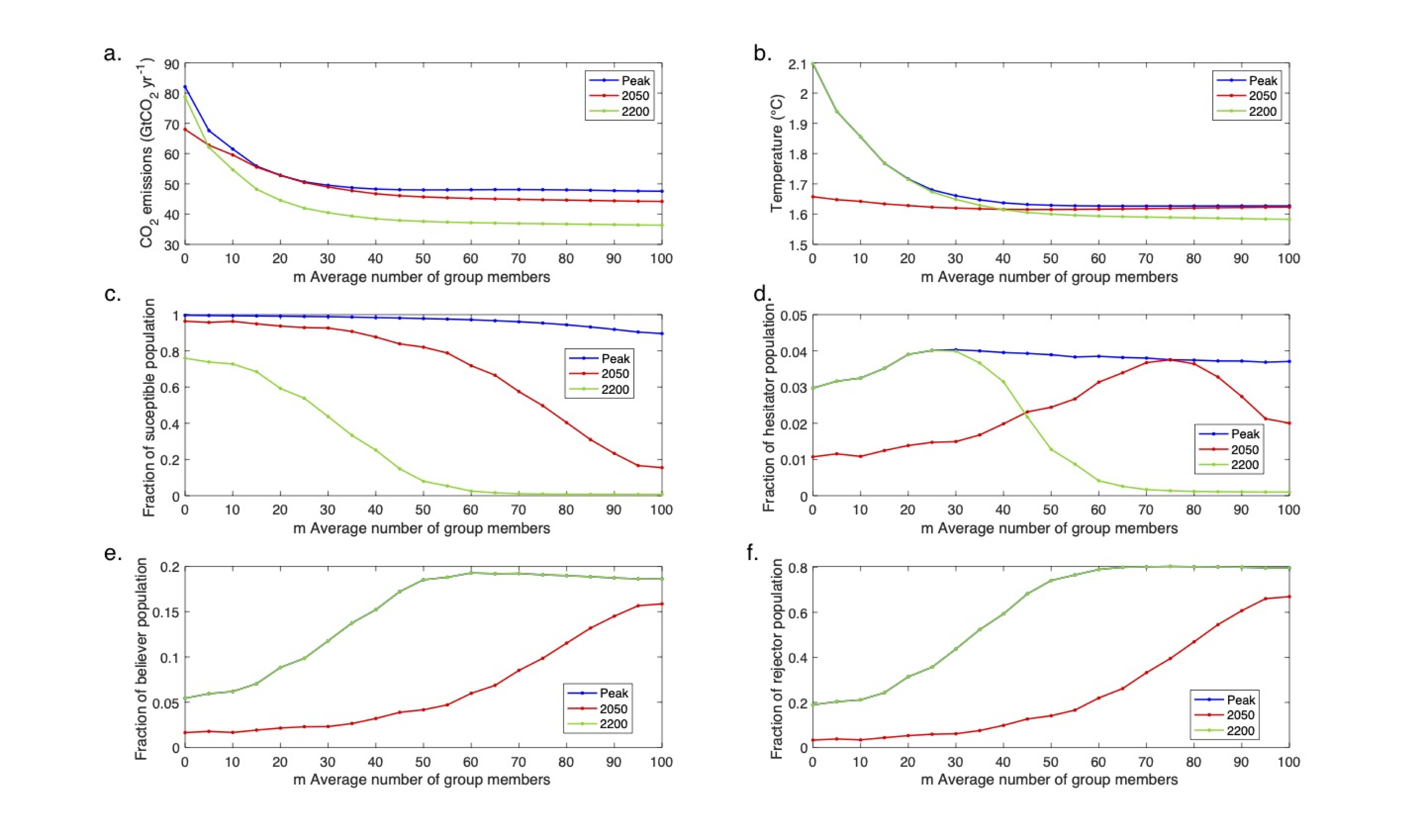

Supplement: S7 Fig — Univariate sensitivity of (a) CO2 emissions, (b) temperature, (c) susceptible, (d) hesitator, (e) believer, and (f) rejector population by varying the parameter m, the average number of group members. (TIF) [file pone.0317338.s007.tif]

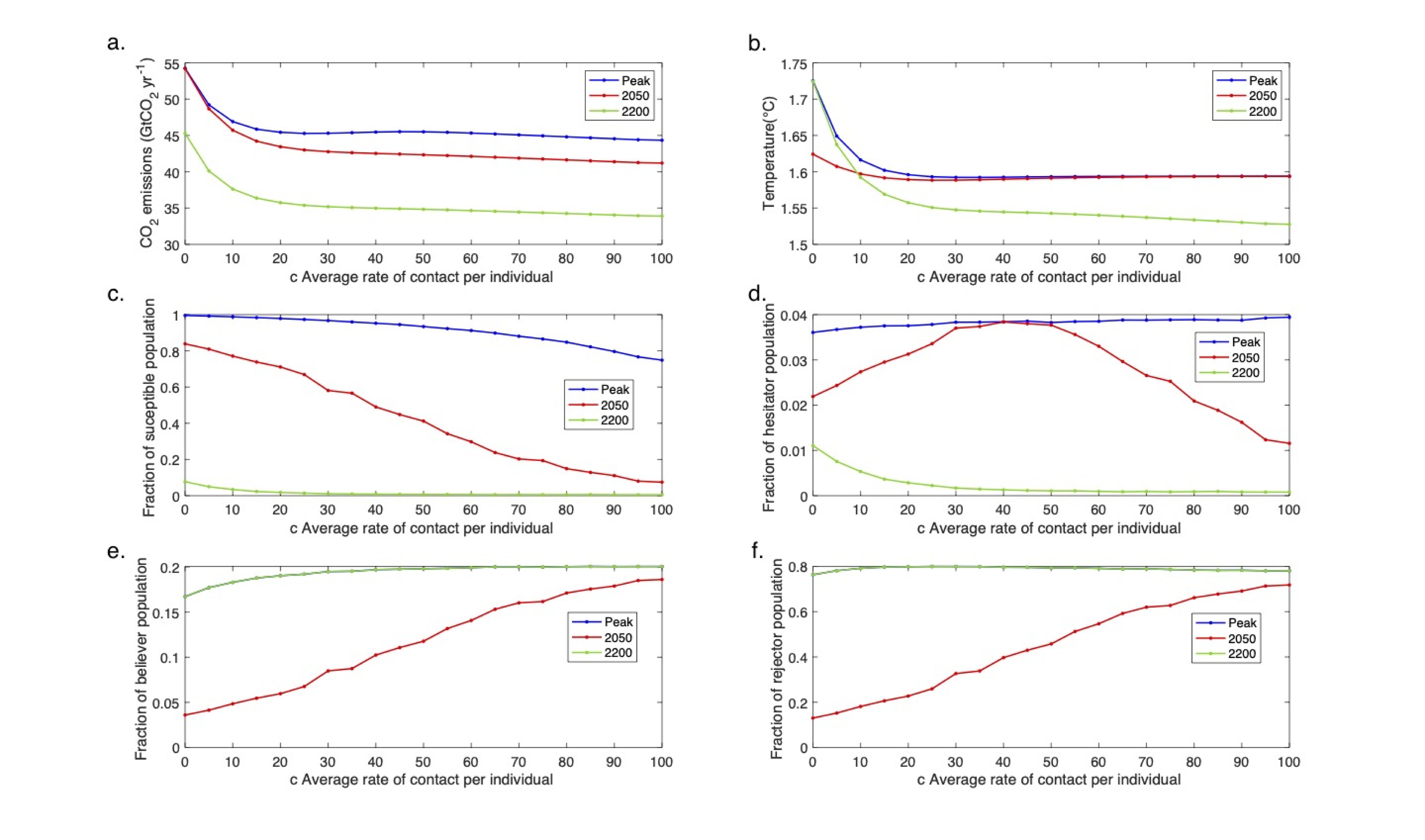

Supplement: S8 Fig — Univariate sensitivity of (a) CO2 emissions, (b) temperature, (c) susceptible, (d) hesitator, (e) believer, and (f) rejector population by varying the parameter c¯, the average number of contact for each individual. (TIF) [file pone.0317338.s008.tif]

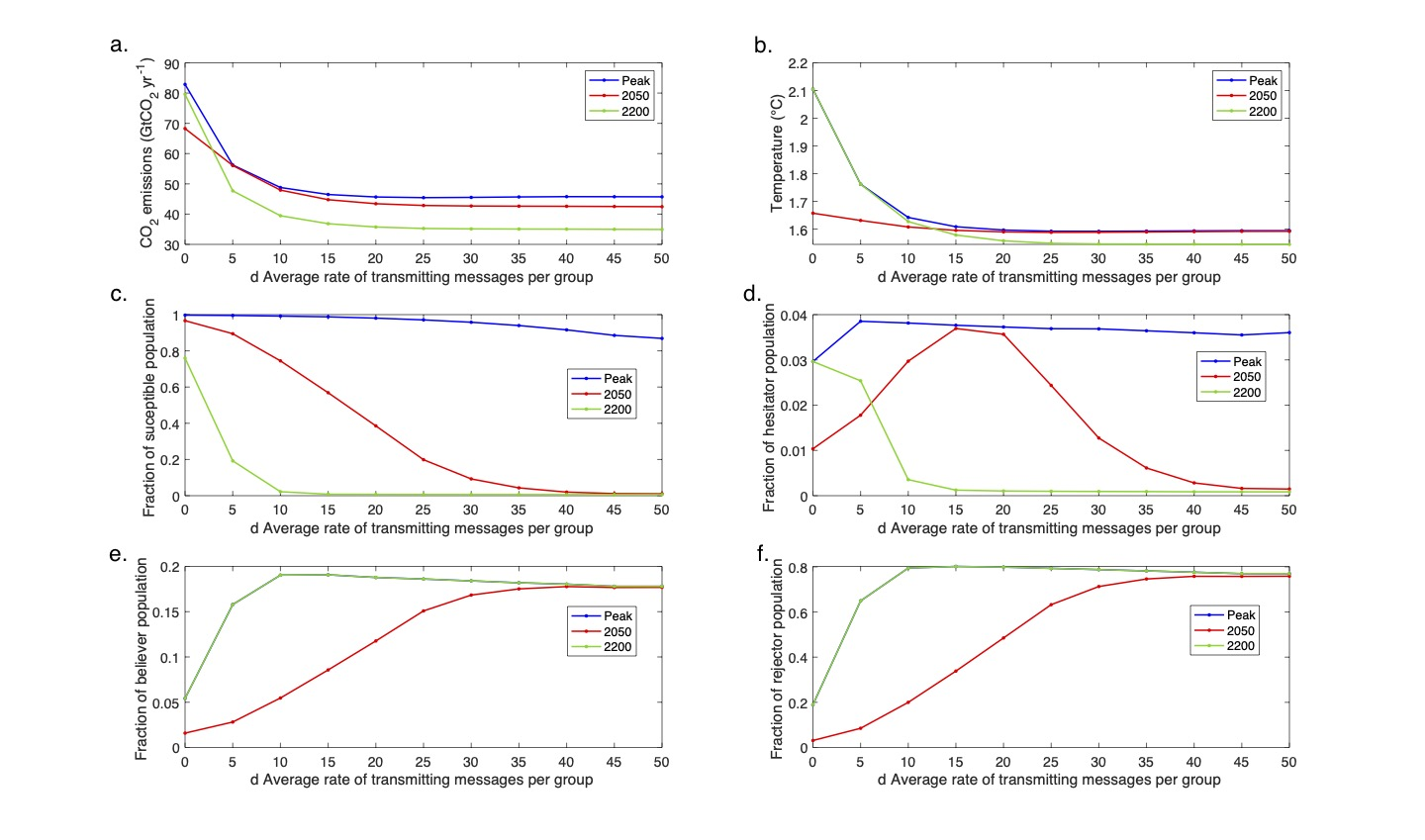

Supplement: S9 Fig — Univariate sensitivity of (a) CO2 emissions, (b) temperature, (c) susceptible, (d) hesitator, (e) believer, and (f) rejector population by varying the parameter d¯- the average rate of transmitting messages per group. (TIF) [file pone.0317338.s009.tif]

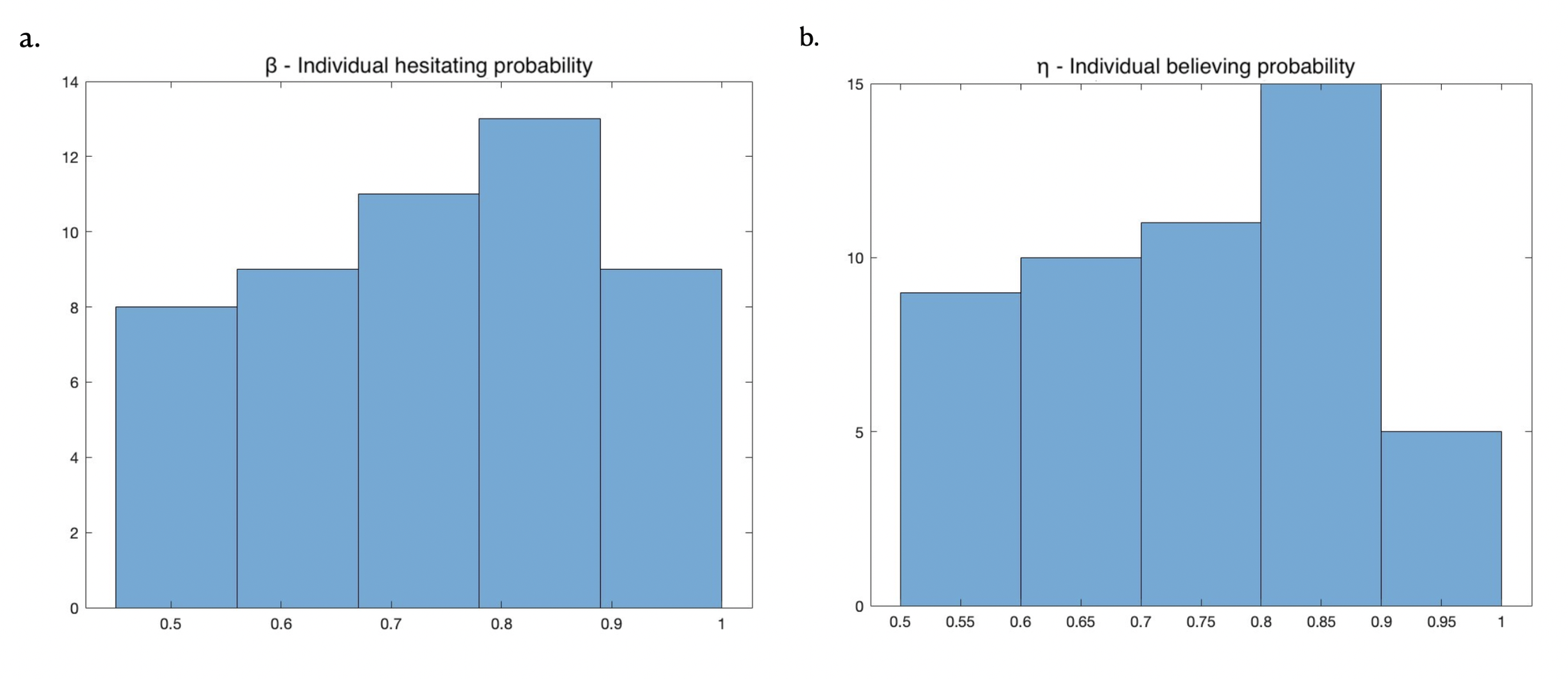

Supplement: S10 Fig — (TIF) [file pone.0317338.s010.tif]

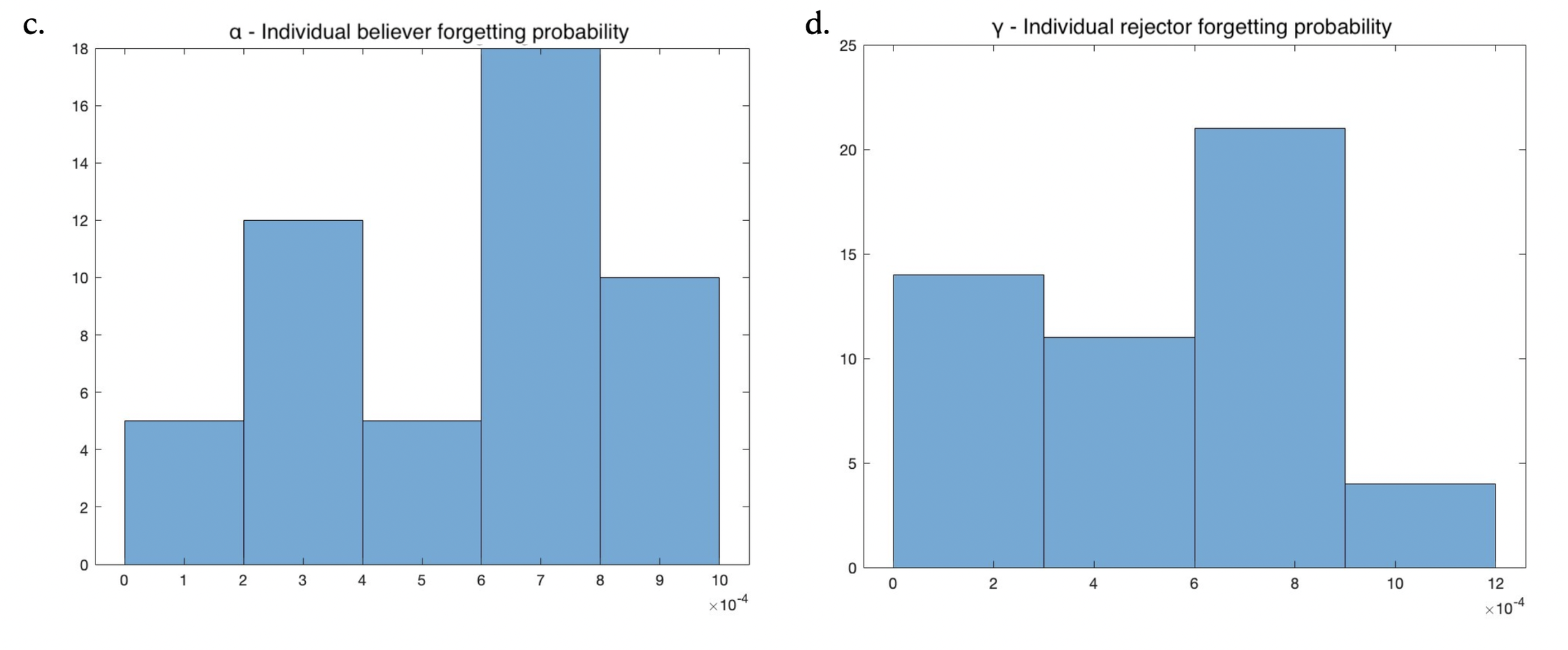

Supplement: S11 Fig — (TIF) [file pone.0317338.s011.tif]

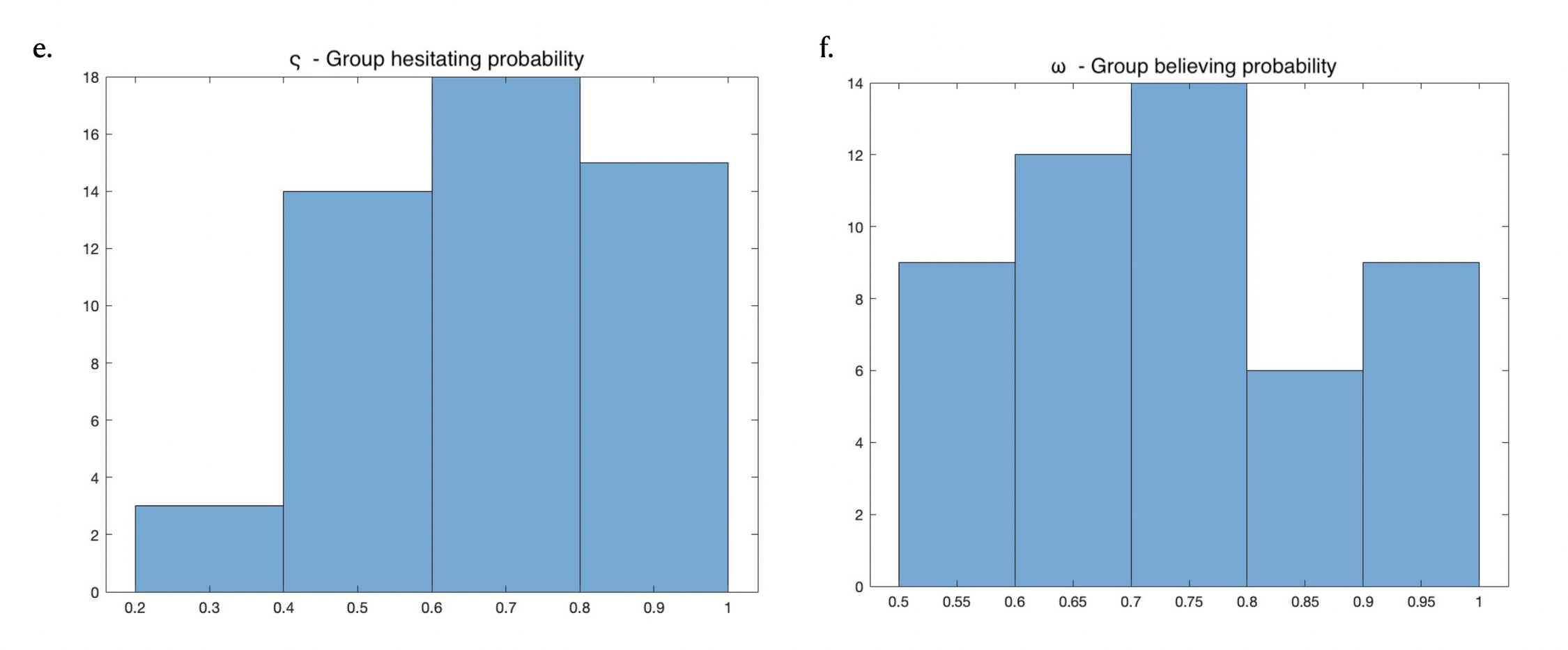

Supplement: S12 Fig — (TIF) [file pone.0317338.s012.tif]

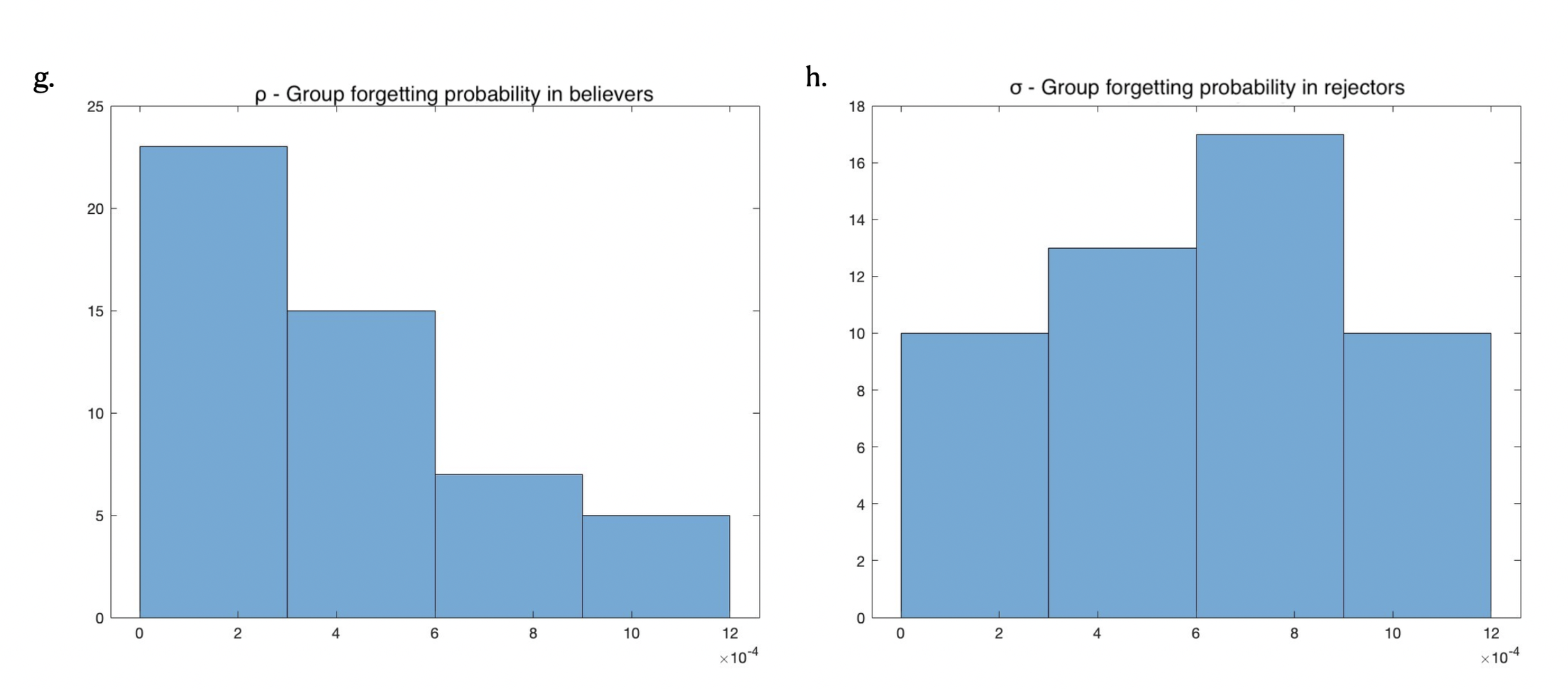

Supplement: S13 Fig — (TIF) [file pone.0317338.s013.tif]

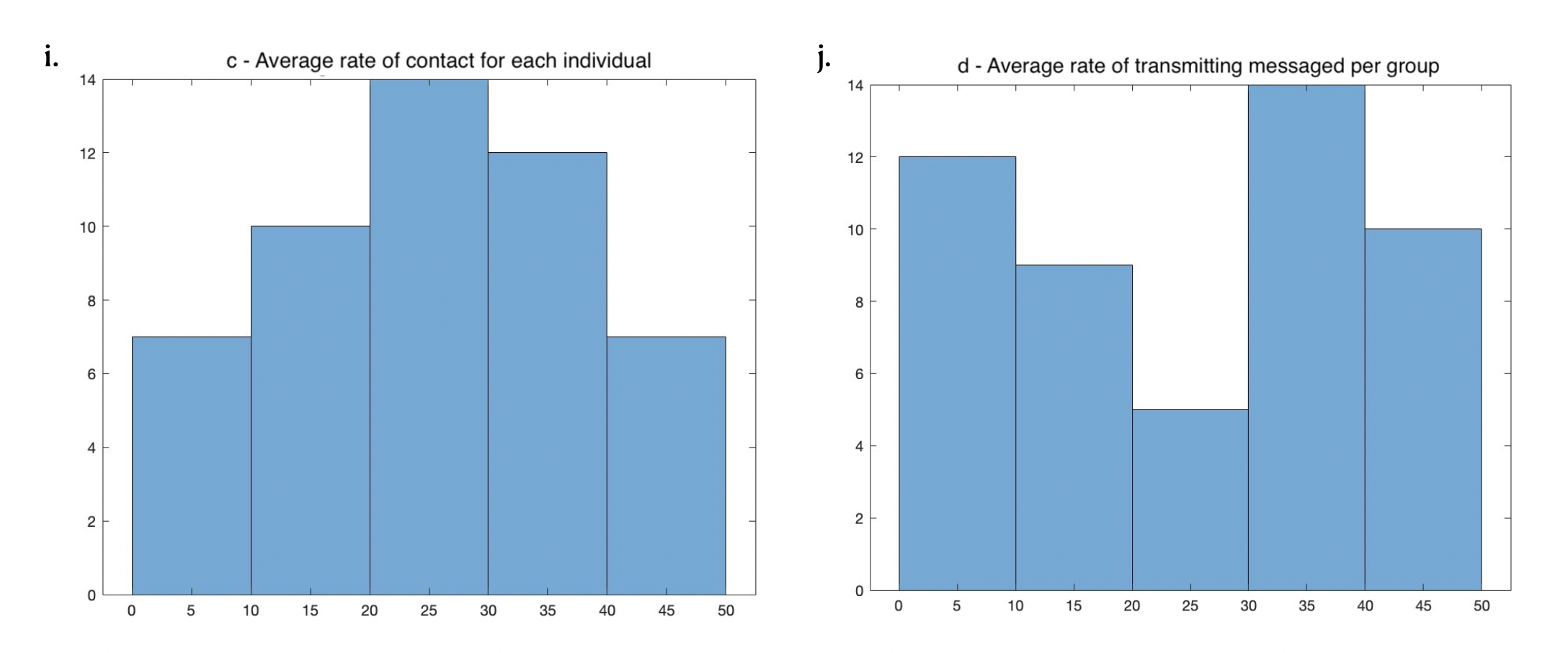

Supplement: S14 Fig — (TIF) [file pone.0317338.s014.tif]

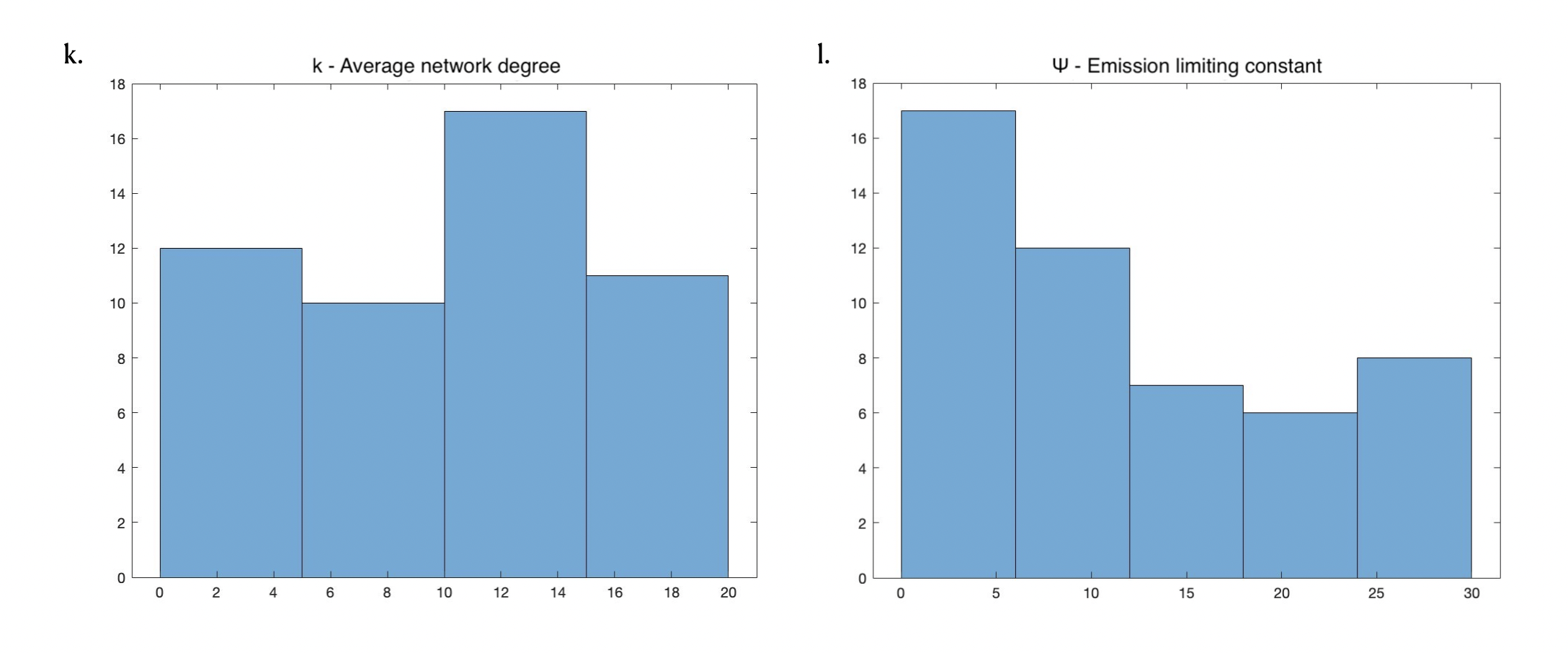

Supplement: S15 Fig — (TIF) [file pone.0317338.s015.tif]

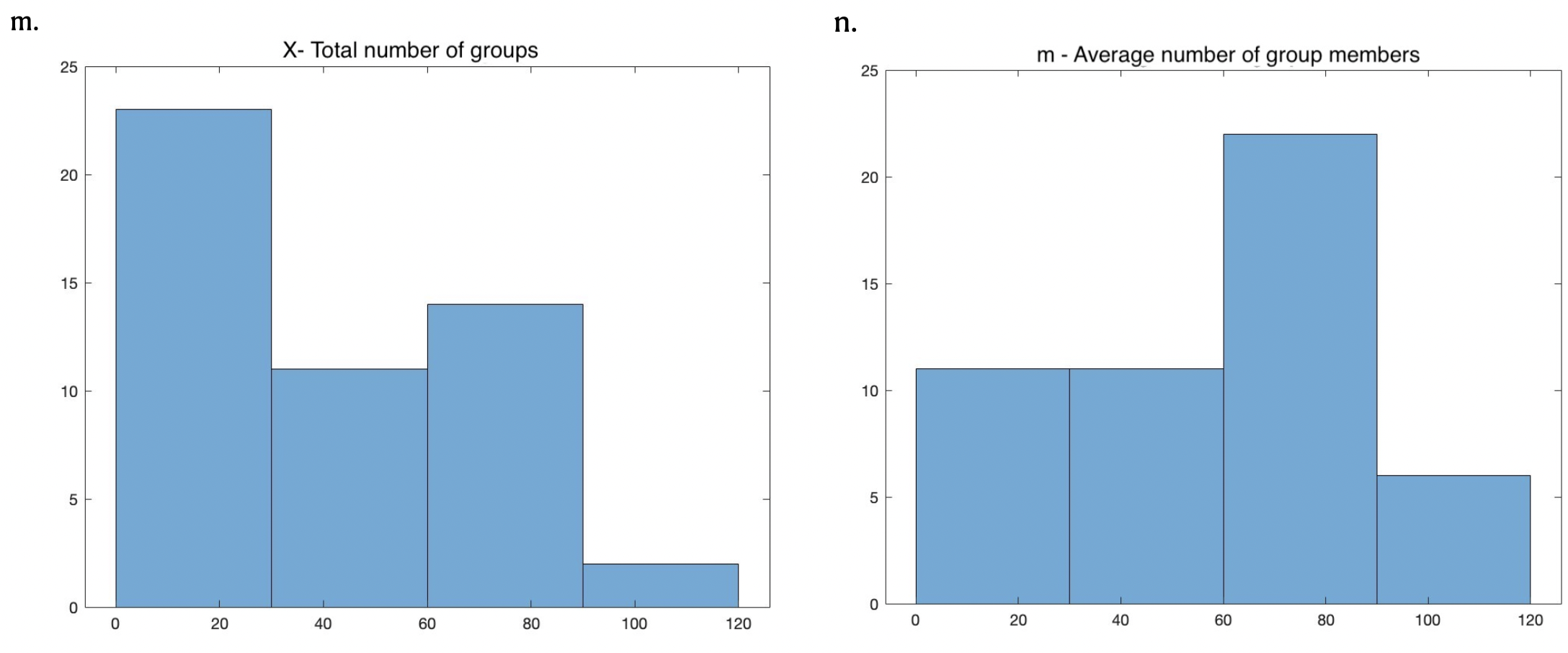

Supplement: S16 Fig — (TIF) [file pone.0317338.s016.tif]

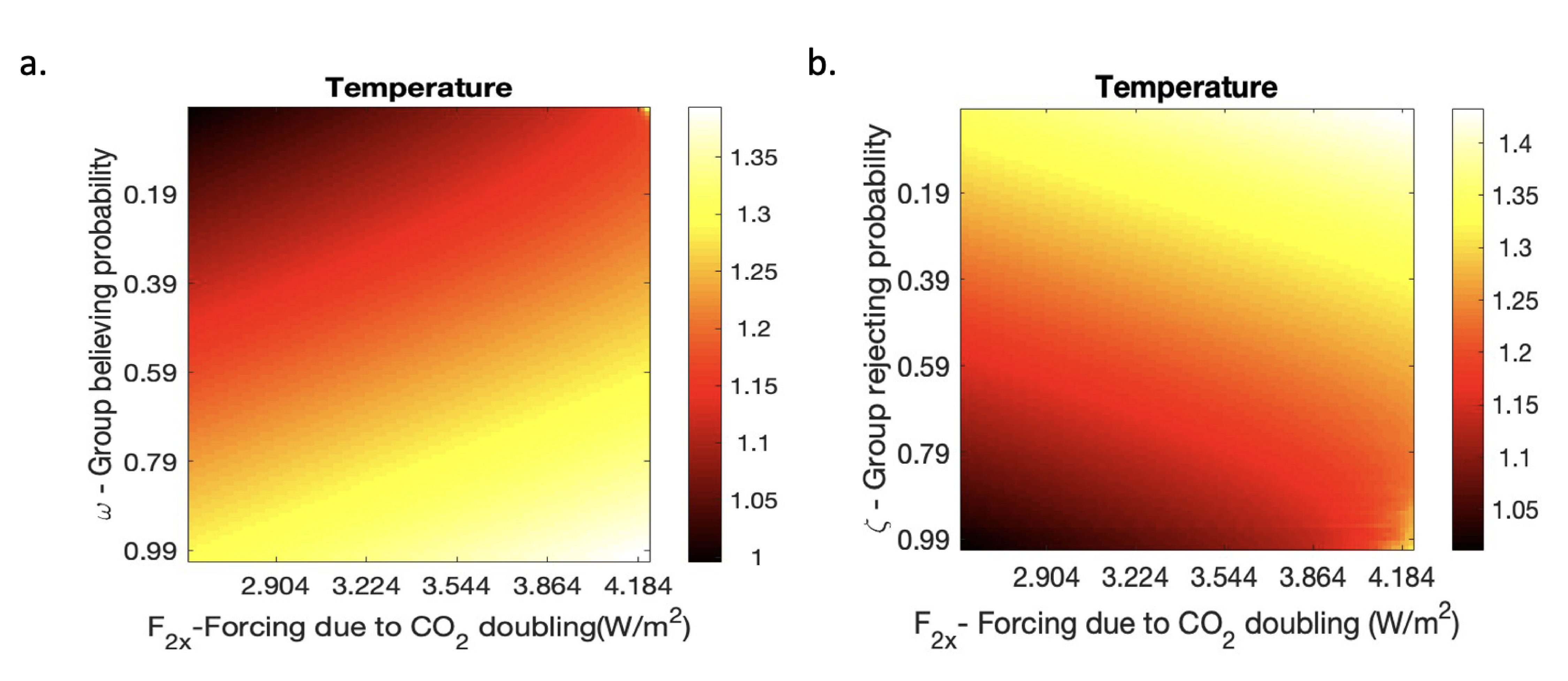

Supplement: S17 Fig — (TIF) [file pone.0317338.s017.tif]

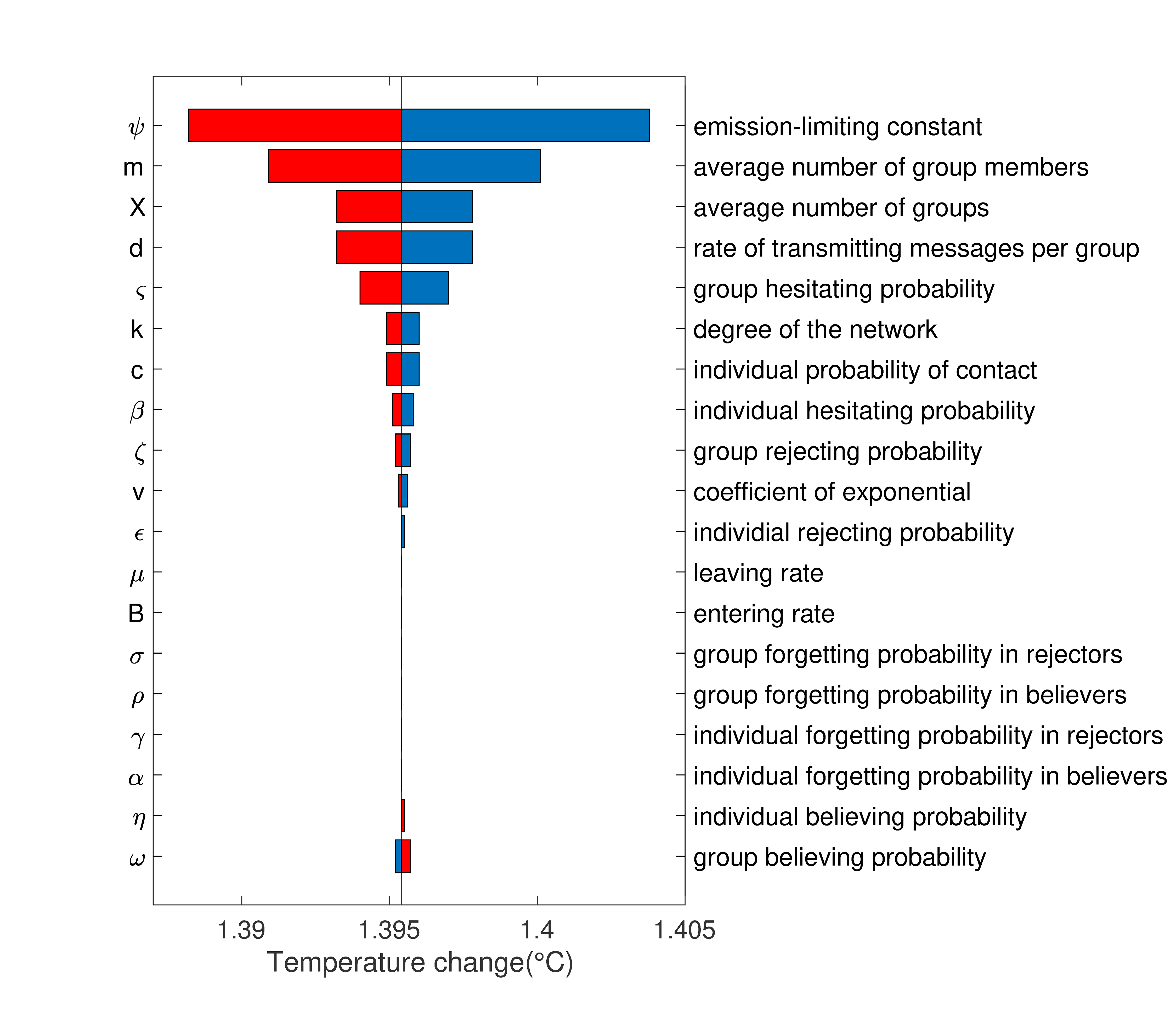

Supplement: S18 Fig — Tornado plot showing the sensitivity of temperature change when parameters are varied individually, with a 10% increase and decrease from the baseline values. The red color represents an increase in the parameter value and the blue color represents a decrease in the parameter value, from the baseline value. (TIF) [file pone.0317338.s018.tif]
