# Supplementary material for: Climate-denying rumor propagation in a coupled socio-climate model: Impact on average global temperature
Source: PLoS One. 2025 Jan 16;20(1):e0317338. doi: 10.1371/journal.pone.0317338 (PMC11737659; doi:10.1371/journal.pone.0317338)
Supplement: S1 Table — (PDF) [file pone.0317338.s019.pdf]

**Parameters**	**Description**	**Values**	**Units**	**Sources**
β	Hesitating probability (individuals)	(0,0.7716,1)	1	calibrated
ς	Hesitating probability (groups)	(0,0.7924,1)	1	calibrated
η	Believing probability (individuals)	(0,0.3857,1)	1	calibrated
ω	Believing probability (groups)	(0,0.3115,1)	1	calibrated
ϵ	Rejecting probability (individuals)	(0,0.6143,1)	1	calibrated
ζ	Rejecting probability (groups)	(0,0.6885,1)	1	calibrated
α	Forgetting probability in believers (individuals)	(0,0.0003,1)	1	calibrated
ρ	Forgetting probability in believers (groups)	(0,0.0007,1)	1	calibrated
γ	Forgetting probability in rejectors (individuals)	(0,0.00009,1)	1	calibrated
σ	Forgetting probability in rejectors (groups)	(0,0.0007,1)	1	calibrated
$\bar{c}$	Average rate of contact for each individual	(1,27,50)	yr^{-1}	calibrated
$\bar{d}$	Average rate of transmitting messages per group	(1,11,50)	yr^{-1}	calibrated
δ	CO_2 dissipation rate	0.06	yr^{-1}	[4,11]
Ψ	Emission limiting constant by rejectors	10	1	calibrated
E_{2021}	Annual global carbon emission in 2021	36.4	$GtCO_2yr^{-1}$	[58]
B	Proportion of individuals entering the network per unit time	(0,0.1730,1)	yr^{-1}	calibrated
μ	Rate of leaving or exiting the network	(0,0.1119,1)	yr^{-1}	calibrated
k	Average degree of the network	(1,5,20)	1	calibrated
X	Total number of groups	(1,40,100)	1	calibrated
$\bar{m}$	Average number of group members	(1,70,100)	1	calibrated
$\bar{G}$	Average group degree of an individual	3	1	calibrated
C_0	Pre-industrial CO_2 level	38.9	$GTCO_2$	[4,11]
F_{2x}	Forcing due to CO_2 doubling	3.74	Wm^{-2}	[28]
q	Thermal adjustment of upper ocean	0.41	$KW^{-1}m^2$	[28]
d	Thermal equilibrium for upper ocean	4.1	yr	[28]
v	Coefficient of exponential	0.35	C^{-1}	calibrated
