# Supplementary material for: Climate-denying rumor propagation in a coupled socio-climate model: Impact on average global temperature
Source: PLoS One. 2025 Jan 16;20(1):e0317338. doi: 10.1371/journal.pone.0317338 (PMC11737659; doi:10.1371/journal.pone.0317338)
Supplement: S1 Text — (PDF) [file pone.0317338.s020.pdf]

Supplementary Material

Equilibrium points

The equilibrium points for the coupled system is calculated using *syms* and *solve* in MATLAB. *syms* allows to algebraically solve for equilibrium points using *solve*. This method is beneficial in solving the system of equations without assigning numerical values and in finding general solutions.

The equations for $s'_k, h'_k, i'_{B_k}, i'_{R_k}, C'$ and T' are assigned as symbolic variables and functions to find the equilibrium point, in terms of the model parameters. The system of equations derived symbolically yields only one set of values for $s'_k, h'_k, i'_{B_k}, i'_{R_k}, C'$ and T' which is the rumor-free equilibrium $(\frac{B}{\mu}, 0, 0, 0)$ where the system gets decoupled.

Using the Lyapunov function $V(t) = k_1 h_k(t) + k_2 i_{B_k}(t)$ where $k_1 = \frac{1}{((\eta(T)+\varepsilon)k\frac{\sum_j (p(j|k))}{p_j} + \bar{G}\bar{m}\omega(T) + \bar{G}\bar{m}\zeta - \mu)}$ and $k_2 = \frac{1}{(\alpha k \frac{\sum_j (p(j|k))}{p_j} + \bar{G}\bar{m}\rho + \mu)}$ [1], hence $V^{'}(t) = k_1 h^{'}_k(t) + k_2 i'_{B_k}(t)$. Substituting the values of k_1, k_2, h'_k, i'_{B_k}, the equation for $V'(t)$ simplifies to the form:

$$V^{'}(t) = (\frac{B(\beta\bar{c}k\sum_k \frac{(p(k/k))}{p_k} + \bar{G}\bar{m}\bar{d}\varsigma)(\eta(T)\bar{c}k\frac{\sum_j (p(j/k))}{p_j} + \bar{G}\bar{m}\bar{d}\omega(T))}{\mu((\eta(T)+\varepsilon)k\bar{c}\frac{\sum_j (p(j/k))}{p_j} + \bar{G}\bar{m}\bar{d}\omega(T) + \bar{G}\bar{m}\bar{d}\zeta + \mu)(\alpha k\bar{c}\frac{\sum_j (p(j/k))}{p_j}) + \bar{G}\bar{m}\bar{d}\rho + \mu)} - 1$$

$$(1 + \frac{\eta(T)k\frac{\sum_j (p(j|k))}{p_j} + \bar{G}\bar{m}\omega(T)}{\alpha k\frac{\sum_j (p(j|k))}{p_j} + \bar{G}\bar{m}\rho + \mu})h_k(t)$$

Checking the value of the terms in the above equation by substituting the parameter values shows that the value of the first term with the combination of parameters is always < 1, and the second term with the combination of parameters is always > 1. Hence, $V'(t)$ is strictly negative, ensuring no equilibria other than the rumor-free equilibrium exists.

Stability analysis for the coupled model

To prove the stability of the system of equations (1.1) for the coupled model, consider the rumor-free equilibrium (RFE), $E_0 = (\frac{B}{\mu}, 0, 0, 0)$. The coupled model becomes decoupled in the case of RFE when there are no hesitators, believers, and rejectors in the population. Hence, the stability simplifies to be the same as for the rumor model.

Consider the Lyapunov function $V(t)$ to be $V(t) = k_1 h_k(t) + k_2 i_{B_k}(t)$ by assuming that $k_1 = \frac{1}{((\eta+\varepsilon)k\frac{\sum_j (p(j|k))}{p_j} + \bar{G}\bar{m}\omega + \bar{G}\bar{m}\zeta - \mu)}$ and $k_2 = \frac{1}{(\alpha k\frac{\sum_j (p(j|k))}{p_j} + \bar{G}\bar{m}\rho + \mu)}$ [1].

$$V^{'}(t) = k_1 h^{'}_k(t) + k_2 i'_{B_k}(t)$$

Substituting for $h^{'}_k(t)$ and $i'_{B_k}(t)$ from equation (1.1) and simplifying,

$$V^{'}(t) = (R_0 - 1)[1 + \frac{(\eta k\frac{\sum_j (p(j|k))}{p_j} + \bar{G}\bar{m}\omega))}{(\alpha k\frac{\sum_j (p(j|k))}{p_j} + \bar{G}\bar{m}\rho + \mu)}]h_k(t)$$

after substitutions and simplifications [1].

According to the Lyapunov condition, when $R_0 < 1$, $V^{'}(t) < 0$ for all $t \geq 0$, and $V^{'}(t) = 0$ only if $h_k(t) = 0$. Therefore, establishing that the rumour-free equilibrium is globally asymptotically stable.

References

1. Myilsamy K, Kumar MS, Kumar AS. Optimal control of a rumor model with group propagation over complex networks. Int J Mod Phys C. 2021;32(03):2150035.
